# Review on Electrospun Nanofiber-Applied Products

**DOI:** 10.3390/polym13132087

**Published:** 2021-06-24

**Authors:** Fatirah Fadil, Nor Dalila Nor Affandi, Mohd Iqbal Misnon, Noor Najmi Bonnia, Ahmad Mukifza Harun, Mohammad Khursheed Alam

**Affiliations:** 1 Textile Research Group, Faculty of Applied Sciences, Universiti Teknologi MARA, Shah Alam 40450, Selangor, Malaysia; fatirahfadil@uitm.edu.my (F.F.); texiqbal@uitm.edu.my (M.I.M.); 2 Materials Science and Technology, Faculty of Applied Sciences, Universiti Teknologi MARA, Shah Alam 40450, Selangor, Malaysia; noornajmi@uitm.edu.my; 3 Faculty Engineering, University Malaysia Sabah, Kota Kinabalu 88400, Sabah, Malaysia; 4 College of Dentistry, Jouf University, Sakaka 72721, Saudi Arabia; dralam@gmail.com

**Keywords:** electrospinning, nanofibers, nonwoven fabric, applications, applied product

## Abstract

Electrospinning technology, which was previously known as a scientific interdisciplinary research approach, is now ready to move towards a practice-based interdisciplinary approach in a variety of fields, progressively. Electrospun nanofiber-applied products are made directly from a nonwoven fabric-based membranes prepared from polymeric liquids involving the application of sufficiently high voltages during electrospinning. Today, electrospun nanofiber-based materials are of remarkable interest across multiple fields of applications, such as in electronics, sensors, functional garments, sound proofing, filters, wound dressing and scaffolds. This article presents such a review for summarizing the current progress on the manufacturing scalability of electrospun nanofibers and the commercialization of electrospun nanofiber products by dedicated companies globally. Despite the clear potential and limitless possibilities for electrospun nanofiber applications, the uptake of electrospinning by the industry is still limited due to the challenges in the manufacturing and turning of electrospun nanofibers into physical products. The recent developments in the field of electrospinning, such as the prominent nonwoven technology, personal views and the potential path forward for the growth of commercially applied products based on electrospun nanofibers, are also highlighted.

## 1. Introduction

**Figure 1:**
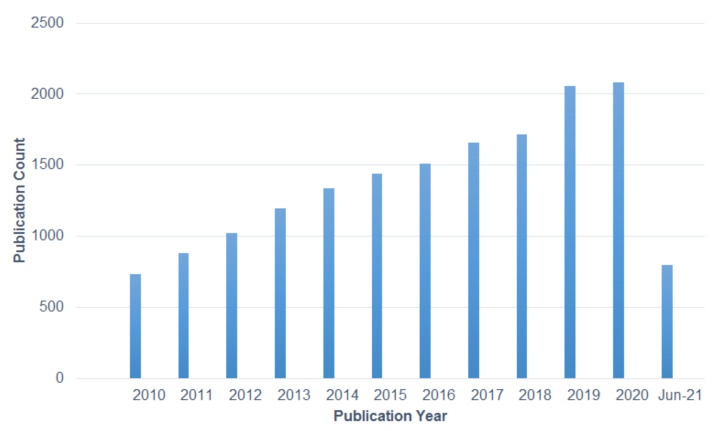
Graph showing the total relevant published articles on electrospun nanofiber-related research between 2010 and June 2021 by research outputs with the search topic “electrospun nanofibers” carried out using the Web of Science online search system as of 17 June 2021.

Nanotechnology is a fast-growing interdisciplinary technology that is concerned with the novel changes and drastic improvements in the properties of materials conjugated to their nanosized structures [1]. The production of electrospun nanofibers has contributed to a new generation of nonwoven fabric-based materials for useful applications in multidisciplinary research areas [2,3,4,5]. The emergence of driven research and the development of electrospinning technology and its electrospun nanofibers is denoted by the increasing publication of related electrospinning research works. Figure 1 presents a survey of the total publications with search topics of electrospun nanofibers between 2010 and June 2021. Quantitative data in the literary study surveys were supported from the Web of Science online search system.

Electrospun nanofibers are derived from electrospinning technology, with exceptional characteristics such as a high surface-to-volume ratio, interconnected ultrafine fibrous structure, high tortuosity, high permeability and lightweight materials. Electrospun nanofibers with diameters as low as 100 nm have continuously attracted broad attention from worldwide researchers, who are ready to move towards a practice-based interdisciplinary approach in a variety of fields [6,7,8,9,10]. This path of inventions and discoveries of electrospinning technology was started in the early of 1930s by a German inventor, Anton Formhals, with the big idea of processing artificial silk-like fibers. At the beginning of the 20th century, silk was very popular as an expensive material and high-end textile. It was thus an interesting search to find a replacement material that was inexpensive, and Formhals was actually the beginning to finding a way to generate threads from a dissolved solid in an electrical field. His series of 22 patents, associated with the progress of the electrospinning process, however, could not compete against the commercial large-scale fiber-spinning techniques in the past decades; thus, this delayed their further development for years [11]. Though the technique of electrospinning is quite old, it has undergone rapid development in recent years after the breakthrough of the exceptional nanostructural characteristic of nanofibers produced from electrospinning in the late 1960s [12,13]. At present, electrospinning is widely used to produce one-dimensional (1D) nanostructures of polymer fibers and is known as the prominent processing technique to generate an interconnected fibrous web from solutions of different polymers and polymer blends that relies on a high-voltage environment.

**Figure 2:**
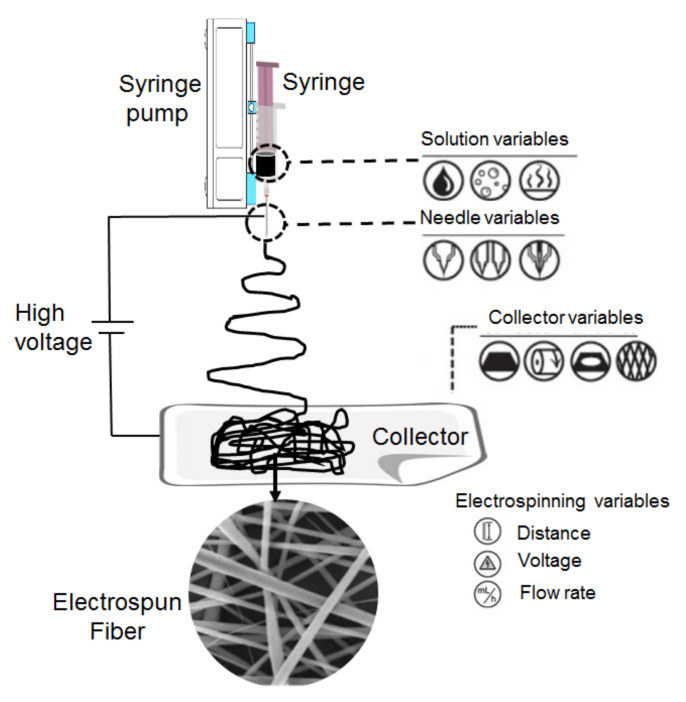
Schematic representation of a vertical electrospinning setup.

Electrospinning is a versatile technique and is evidenced by the accessibility to tailor electrospun fiber production into a rich variety of fiber morphologies. These electrospun nanofibers can be designed into various morphological structures, such as core shell hollow, porous, deposited in the aligned or a random orientation of nanofiber mesh, using a single or blended copolymer, with the incorporation of additive materials, subjectively [14]. The versatility of electrospinning technology has accelerated the progressive development of a wide range of nanofiber-based products, with tailored compositions, dimensions and morphologies to carry out various functionalities [15,16,17]. Figure 2 shows a schematic representation of an electrospinning setup. A standard laboratory electrospinning machine prerequisite was equipped with a high-voltage source, precision syringe pump and conductive collector, separated at an appropriate distance. The power supply was attached to each charged spinneret and, also, the grounded collector. Spinning solutions filled in the syringe were loaded onto the metal spinneret and subjected to high-voltage charges. The induced electrical forces to surpass the surface tension of the charged liquid followed by the transformation of the pendant drop shape of the liquid meniscus into a “Taylor cone” shape. The whipping and bending instability upon charging the liquid produces a jet of ultrafine fibers before reaching the collector as electrospun fibers [18,19,20,21]. Upon the electrospinning process, the deposited nanofiber mesh is usually collected as sheets of 10–30-mm-thickness, on top of a conductive substrate. The sheet of nanofibers mesh usually needs to be removed from the conductive substrate and be practicable to be applied in their next applications.

The growing interest in electrospinning technology has paved the way to their advanced development by offering the controllable inclusion of the nanoparticles into these nanosized fibers through the blending of the nanoparticles-polymer solution prior to the spinning process. The simplicity, high efficiency, low cost and high reproducibility of nanofibers have categorized electrospinning as the potent fabrication technique of fibrous structured materials in nanometer sized [22,23]. In line with prior published studies, the exploration for practical applications of nanofibers primarily focused on filtration, functional textile, biomedical and electronic devices [11,24,25,26]. Researchers have published a number of review articles on electrospinning parameters, characterization, and electrospun nanofiber applications [14,18,22,25,27,28,29,30,31,32,33,34,35,36]. However, these past research has not extensively addressed the commercialization aspect of the fibers and their commercial nanofiber-based product. Hence, this article aims to present an overview for summarizing the current progress on the manufacturing scalability of electrospun nanofibers and the commercialization of electrospun nanofibers products by the dedicated companies globally. The scope of this review will cover the recent progress on the nanofiber fabrics manufacturing by electrospinning, the challenge faced in quality control and manufacturing scalability, the latest finding on nanofiber-based products and an outlook on what the future may hold for electrospun nanofibers as prospect resources are well highlighted in this review article.

## 2. Electrospinning Fundamentals

Electrospinning has been acknowledged as an ancient nanotechnology rediscovered for modern demands, being intensely practiced in both laboratories and industries around the whole world. In order to be able to manufacture nanofiber-based fabrics from electrospun nanofibers in mass production, the need for proper control over the electrospinning processing is very crucial. Electrospinning involves a very simple and cost-effective method, yet is an intricate process that crucially depends on several main processing and technical parameters [37,38,39,40,41]. The quality of electrospun fibers is enormously governed by the following multiple influential variables:

1. Solution variables: concentration, degree of polymerization, viscosity and surface tension of the polymer.
2. Needle variables: mono-axial, co-axial, tri-axial and needle-free.
3. Collector variables: stationary collector, drum rotary collector and rotational speed.
4. Electrospinning variables: voltage, feed rate, distance, temperature and humidity.

An optimized concentration of polymer solution is needed in order to control the formation of electrospun fibers, to be free from defectiveness such as beaded fibers or sprayed droplets. It is mentioned that as below the optimized spinning solution concentration was prepared, the polymer chain entanglements are insufficient to stabilize the Coulombic repulsion within the ejected jet, leading to the formation of sprayed droplets [42,43]. While the spinning solution feed rate which is usually applied during the electrospinning process is in the range of 0.5–2.0 mL/h. The feed rate and the voltage are actually worked simultaneously in order to produce a stable Taylor cone shape, to avoid droplet formation. For the fabrication of polysulfone electrospun fibers, Yuan et al. (2004) preferred a lower feed rate for spinning, since a greater volume of solution drawn will only take a longer time for solvent to dry. Otherwise, the remaining residual solvents may cause the fibers to fuse as they hit onto the collector, consequently leading to the imperfection of the fibrous structure [16].

Another important concern in regard to ecological impact is related to the use of organic solvents in electrospinning. The importance of this factor not only denoted the safety during the spinning handling but also the quality of produced nanofibers due to the entrapped of residual solvent. A precise monitoring to minimize residual solvents is vital for clinical medicine studies as compared to other applications. The use of safe, environmentally benign solvents in the electrospinning should be developed, to decrease the large consumption of volatile organic solvents.

Besides, the formation of uniform diameter sizes of electrospun nanofibers is driven by the surface tension properties of polymer solution. Zhang (2011) clarified that by using a smaller internal diameter of a needle, the size of the charged droplet at the tip is reduced, consequently increases the surface tension of the charged droplet. As the surface tension is increased, extra energy is required to overcome the force. As a result, the decrease in the acceleration of the jet will give more time for the droplet to be stretched into a smaller size of fibrous form before deposited on the collector [44]. On the other hand, Taylor did mention the minimum applied voltage for the initiation of the electrospinning process to occur is at 6 kV. Further study by Elsabee et al. (2012) revealed that by lowering the surface tension of spinning solution helps the electrospinning process to be initiated even at lower electric fields [45]. It is also reported that the addition of nanomaterials such as carbon nanotubes (CNTs) has been facilitating in reducing the surface tension of the spinning solution, also aid to electrical conductivity, thus allowing the use of low high voltage applied for the spinning process [46,47,48,49]. Another work reported by Nayak et al. (2012) has demonstrated the use of conductive additives such as sodium oleate (SO) and sodium chloride (NaCl) to aid in stabilizing the electrical conductivity during the spinning process. Their finding reported the increment in the electrical conductivity was greater with the introduction of NaCl, due to the smaller ionic size as compared to SO. The hydrophobicity of as-spun fibers was marginally decreased with the addition of SO and NaCl [50].

### From Laboratory to Industrial Scale

**Figure 3:**
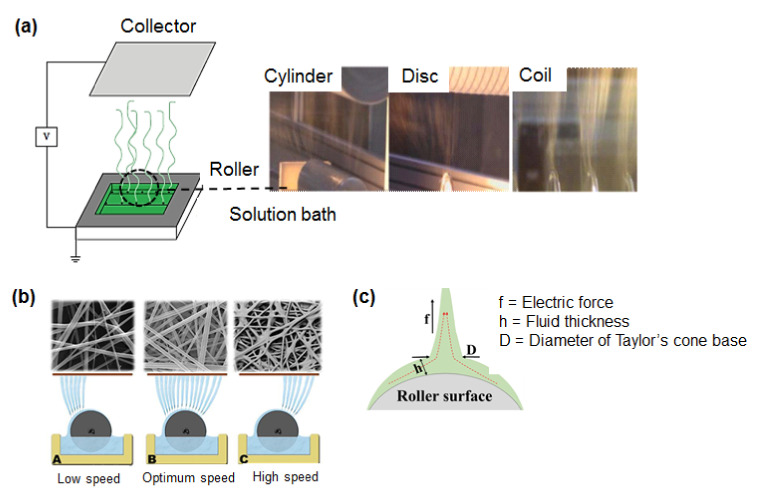
(**a**) Illustration of a needle-free electrospinning system. (**b**) Effect of the roller speed on the quality of a nanofiber web. (**c**) Schematic diagram of a Taylor’s cone on a roller surface. Figure is adapted and modified from [58].

While electrospinning technology offers an exciting insight in the fabrication of distinctive nanofiber-based materials, a significant hurdle has remained. Current innovative applications and the spinnable fluid limitation are still restricted significantly owing to the inconsistency and instability of electrospinning to assure reliability in processing nanofibers. These existing hurdles boost a robust urge in improving manufacturing efficiency, in order to deal with the related limitation issues involving macro-scale production, process stability and reproducibility in the manufacturing steps of electrospinning as described [12,37]. The major challenges faced in the large-scale manufacturing of electrospun fibers are due to their low output (0.01g–2 g/h) owing to the use of single needle nozzles. [51,52,53,54,55,56]. According to Persano et al. (2013), the upgrading and mass-scale competency demonstrated that needle-free electrospinning systems show off excessive promising possibilities to accelerate the nanofibers production. The combination of centrifugal force with electrospinning is enabling the production of assembly oriented continuous nanofibers below 100 nm has been validated [12]. The benefits of using needle-free electrospinning were also agreed upon by other researchers. Researchers are now established with the development of needle-free electrospinning systems to mass-produce quality electrospun nanofibers to switch to an industrial scale. Research conducted by Niu et al. (2012) highlighted the improvement in the rate of nanofibers production. Through their research, they have successfully constructed a coil-based, needle-free electrospinning system, which has higher fiber productivity, with a width up to 2 m with polyacrylonitrile (PAN) fibers production rate of 23 g/h [57]. The configuration of needle-free electrospinning systems with various collector designs used in scale-up electrospinning reactors, quality of nanofibers at various roller speeds and Taylor’s cone shape of spinning fluid at roller surface is illustrated in Figure 3a–c, respectively. Besides, the intersection understanding of both theoretical modeling and real-time processes to optimize the processing variables will be useful for processing clarifications toward increasing production rate [12].

Based on previous studies, it is largely demonstrated that environmental conditions are an essential stimulus to the outcome of charged dispersed fibers jets. It is reported that a substantial variation in the fiber properties is directly affected by the minor changes in the environment. To resolve the matter, a great improvement of electrospinning systems has been proposed to incorporate the proprietary climate-controlled electrospinning systems with promising temperature and humidity regulators. For instance, IME Technologies provides a laboratory-scale system consisting of an electrospinning compartment and a control cabinet inclusive of the ventilation system, water filtration and regulator with specific temperature and relative humidity accuracy [12,59].

Past studies also have highlighted that electrospun nanofibers are also seldom handled without additional support since their physical structure appears to be soft and thin similar to facial tissue. This delicate feature is an inherent limitation for the electrospun nanofibers which are meant to hold or absorb significant volumes of solids or liquids. As a result, electrospun nanofibers are usually strengthened by turning into a composite, with the embedment of other material as a support, in order to enhance their structure [60,61]. Researchers are also looking into another technique to improve the limitation of sheet-like assembly of electrospun nanofibers through innovative methods for preparing a stable 3D electrospun nanofibers structure [62]. There are few methods for the fabrication of 3D electrospun nanofibers scaffolds such as multi-layering electrospinning [63], liquid and template-assisted electrospinning [64], porogen-incorporated electrospinning [65] and post-treated electrospinning [66]. Post-treated electrospinning via gas foaming offers a versatile way of expanding the 2D membrane of electrospun nanofibers to form a 3D scaffold without excessive application of force. Jiang et al. (2015) reported the usage of NaBH_4_ as the foaming agent in expanding 2D electrospun polycaprolactone (PCL) nanofibers. A higher concentration of foaming agent was found to significantly increase the thickness of the scaffold. This post-treated method was able to produce 3D scaffolds and reduce the risk of breaking the interconnected fibers structure [67].

Electrospun nanofibers have also been demonstrated to be a good alternative reinforcement candidate for composites due to their good mechanical and flexibility, tunable chemical and physical properties. Electrospun nanofibers have also been recognized as potential revolutionary additives for composites as they possess tremendous physic-chemical and flexibility. In spite of holding voluminous idyllic features for the function as good nanofillers, the previous work in using electrospun nanofibers as potential reinforcements are scarcely reported. The probable cause of the limitation lies in their typical type of nanofibrous architecture with which they can be fabricated. Through advanced progress in electrospinning technology by fabricating three-dimensional structures and nanofibrous yarns, the application of the electrospun nanofiber-based fabric can be more considered [68,69].

With the optimization of the production rate of electrospun nanofibers, along with methods and equipment to characterize and control the process output, should accelerate the applications of nanofibers into new applied products. The low basis weight, small fiber diameter and pore size, high surface area and choice of fibers chemistry are important tools in the development of such products. Upon the optimized quality control of the electrospinning process, the production of nanofiber-based fabrics from electrospun nanofiber-based material can be widely commercialized. On the alternative side, good compatibility towards substances and tunable release properties have brought to much greater numerous functionalities of the ensuing nanofibers with the aid of using needle-free electrospinning technologies.

## 3. Functional Additives in Electrospun Nanofibers

The incorporation of nanoparticles to impart additional functionalities to the composited nanofibers fabrics via electrospinning process has been extensively studied. Additional materials such as nano-metal oxides, ionic liquids and conductive polymers might be integrated into these nanofibrous structures, with the purpose of providing technical function such as the electrical conductivity properties, stains resistant capabilities and antibacterial characteristics to an engineered fabric. Among the abundance of nanoparticles which has the potential to be imparted into nanofibers fabrics, silver (Ag) and titanium dioxide (TiO_2_) were mentioned as the most used nanomaterials followed by silicon dioxide (SiO_2_), CNTs and zinc oxide (ZnO) [70,71,72,73,74,75], whereas various types of polymers are under study for multiple potential applications. For example, polymers, which are widely used and in textiles applications are polyester, polyamide, PAN and polyethylene oxide (PEO) [76,77]. The combination of both polymer and nanomaterials filler in the composited nanofibers textiles are expected to be light, resilient, mechanically flexible, inexpensive and easy to process [78]. Whereby in the context of food science application, it will be critical to only utilize food grade polymers and harmless, nontoxic solvents in the preparation of nanofibers [79].

However, the use of synthetic polymers has always been an issue in any application. Hence, several studies have introduced natural polymers such as collagen, gelatin, elastin, fibrinogen, chitosan in electrospun based products [77,80,81]. The inclusion of honey as an additive in silk fibroin fibrous matrices to prevent infection and accelerating wound healing are reported by Yang et al. (2017). It has been demonstrated that Manuka honey (MH) has bioactive compounds which possess distinctive properties of anti-inflammatory and anti-bacterial function which is crucial for tissue growth and increase pain tolerance [82]. The preparation of nanofibers from polymeric and nonpolymeric matrices was investigated by Moreta et al. (2020). The use of green additives β-cyclodextrin (β-CD) to form a host-guest inclusion complex with cinnamon oil in PVA/β-CD has been conducted. Their finding showed the formation of electrospun nanofibers with CD-CI crystal with 5 and 10 wt% cinnamaldehyde content [83]. Another recent work by Topuz et al. (2021) highlighted the fabrication of nanofibrous hyper-crosslinked cyclodextrin membranes which can effectively trap pollutants from wastewater. The membrane was produced by exclusively incorporating green resources of highly concentrated CD solutions containing naturally occurring graphitic acid linkers [84].

### 3.1. Nanoparticles Inclusion

The inclusion of nanoparticles into electrospun nanofibers can be proceeded through the blending with the spinning solution prior to the electrospinning process. However, the prerequisite for every possible application for proper dispersion of nanoparticles, will control their interaction with the polymer matrix environment. These interactions will ultimately affect the colloidal stability of the nanoparticles within the polymer matrix [85]. Technically, the dispersion of nanoparticles is mostly being conducted using a physical approach. High intensity ultrasound has been widely used to disperse nanoparticles in a liquid solvent [86,87,88]. A study conducted by Rouxel et al. (2011) reported that appropriate sonication amplitudes influenced the aggregate size of nanoparticles cluster [89]. On the other hand, the use of surfactant to assist the homogenous dispersion of nanoparticles also has been studied. The use of surfactant is favorable in the nano-emulsion such as for the cosmetics formulations. Types of nanoparticles that can be incorporated in the cosmetic face mask such as nano-sized gold and nano-sized silver particles, TiO_2_ and ZnO as well as buckyballs for the long lasting anti-bacterial protection, broad UV range protection and scavenger of free radicals function, respectively [70,71,72,73,74].

### 3.2. Nanoparticles Surface Coating

The sol-gel process is used to create gel-like solutions containing nanoparticles suspension which can be applied onto the surface of textiles as a liquid finish to create nanofibers fabrics with functional properties. The process begins with dissolving nanoparticles in a solvent such as alcohol. Once dissolved, several chemical reactions take place that cause the nanoparticles to establish a network throughout the liquid to form a stable suspension. The network transforms the solution into a colloid prior to a drying process to remove excess solvent from the mixture before it can be used to coat onto the fabrics [90]. When nanoparticle coatings are applied to fabrics, the deposited nanoparticles will form bonds with the fibers of the fabric materials. Fabrics treated with nanoparticle coatings during manufacturing may produce materials that kill bacteria, eliminate moisture and odor, and prevent static electricity. Besides, the usage of ionic liquid as a finishing layer on electrospun nanofibers surface has also been studied by Datta et al. (2015). Their research finding reported that electrically conductive electrospun polymer which has been generated with the trapping room temperature ionic liquids (RTILs) making them as possible nano-textiles for energy storage applications with the thermoelectric (TE) activity from Seebeck coefficient value of up to 17.92 μV/K [91].

The previous studies listed above indicate the current development related to the nanofiber-based fabrics production from electrospun polymer nanofibers via electrospinning technique. The optimum working factors in assisting spinning are crucial, such that to induce the spinning process by promoting polymer entanglement and the adjustment of solution viscosity and surface tension of the spinning solution, and promote rapid evaporation upon ejection of the polymer jet from the syringe. The addition of nanomaterials that promote entanglement can help overcome the stipulated hurdles in the spinning process. Once a suitable nanoparticle–polymer blend system has been identified, processors should select suitable blend ratios, overall polymer concentrations in the solvent, and optimize the overall processing conditions.

## 4. Electrospun Nanofiber-Applied Products

Insight on the new interesting applications of electrospun nanofiber-based materials in several product applications of filtration, defense and protection garment, medical dressing, home furnishing, food packaging and cosmetics are discussed. An overview of the nanofiber-based products for research and commercialization is highlighted in the next following sub-sections.

### 4.1. Filter Media

To date, environmental issues relating to air and water pollution have gained great attention due to the increasing concerns on human health. In addition, clean water and sanitation have become one of the world agendas which have been highlighted in the 17 Sustainable Development Goals (SDGs) by the United Nations. One of the alternative ways in treating contaminants from air or water is by filtration. Filtration can be described as separating dispersed particles from a dispersing fluid by porous media. The dispersing medium can be a gas/gas mixture, air or a liquid [92]. In filtration, porous media such as membrane is used to separate dispersed particles from any medium [93,94,95]. Membranes are relatively thin, semipermeable and flexible sheets, which are made by several methods including electrospinning to produce assorted nonwoven membrane structures [14,15,16,17]. An accumulation of randomly oriented nanofibers from electrospinning produces a typical porous membrane in a nonwoven orientation form. The electrospun membrane exhibits a high surface area-to-volume ratio, high porosity and interconnected porous structures that are suitable for microfiltration or ultrafiltration [96]. Given these outstanding properties, the electrospun nanofibrous membranes are of great interest by researchers to use the membranes as air filters.

**Figure 4:**
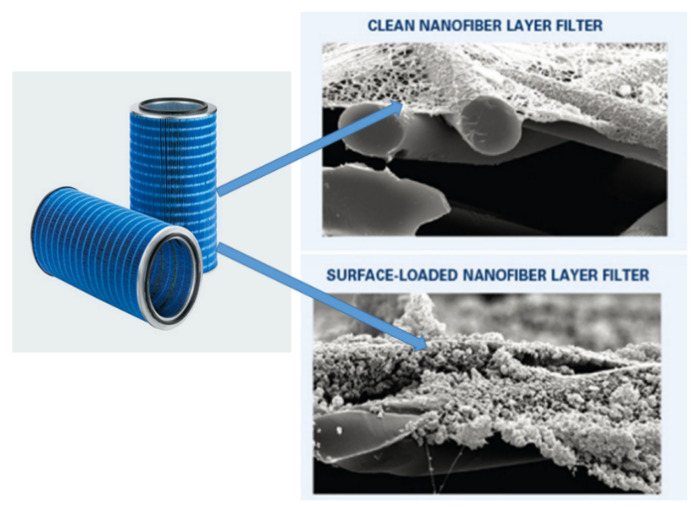
Ultra-web ^®^ filter media by the Donaldson company. Figure is adapted and modified from [97].

Ultra-web ^®^ technology is a commercial air filter made of electrospun nanofibrous membrane. The filter was introduced by Donaldson, a company based in Minneapolis, USA. The deposition of electrospun web on a substrate helps the Ultra-web ^®^ filter media (Figure 4) to trap submicron dust particles on the filter surfaces [97].

Heikkila et al. (2008) verified the filtration efficiency of electrospun polyamide 66 membrane using submicron particles of approximately 0.16 µm. The observation highlighted that the spunbonded polyamide 66 on the nonwoven substrates has significantly enhanced the filtration efficiency [98]. The ability of electrospun membranes to filter fine particles was further enhanced by surface modification of the electrospun membrane. For instance, Huang et al. (2019) used solvent vapor annealing (SVA) treatment to create nano wrinkled fiber surfaces on the poly (ε-caprolactone)/polyethylene oxide (PCL/PEO) blend membrane for effective filtration of particulate matter (PM) 2.5 with high removal efficiency under highlight polluted conditions. In addition, the SVA-treated PCL/PEO air filter mat demonstrated a facile and sustainable preparation process with an outstanding degradation performance than the commercial masks [99]. A study done by Liu et al. (2015) has found that the micro-structured of air filter made of electrospun polyacrylonitrile (PAN) nanofibers able to collect more than 95% of PM2.5 in an urban area with 90% transparency [100].

Another method that can improve the filtration properties of electrospun membrane is by adjusting the electrospun fiber diameter. As reported by Molaeipour et al. (2015), smaller diameter fibers exhibited greater than 10% filtration efficiency than larger diameter fibers. However, the results contradict to the observation reported by Bien and co-workers. The authors found that the filtration efficiency was not affected by the electrospun fiber diameter, but was affected by the membrane thickness. [101]. The results met in agreement with Molaeipour et al. (2015), where the authors reported the double layer of electrospun cellulose acetate membrane showed higher filtration efficiency than the single layer of the membrane [102]. Similar findings were found by Matulevicious et al. (2016), where the multi-ply nanofiber mat gave higher filtration efficiency for 100 nm and 300 nm particles than the single-ply nanofiber mat [103]. An optimal thickness of electrospun membrane for air filtration was about 0.02–0.07 g/m^2^ [104].

**Figure 5:**
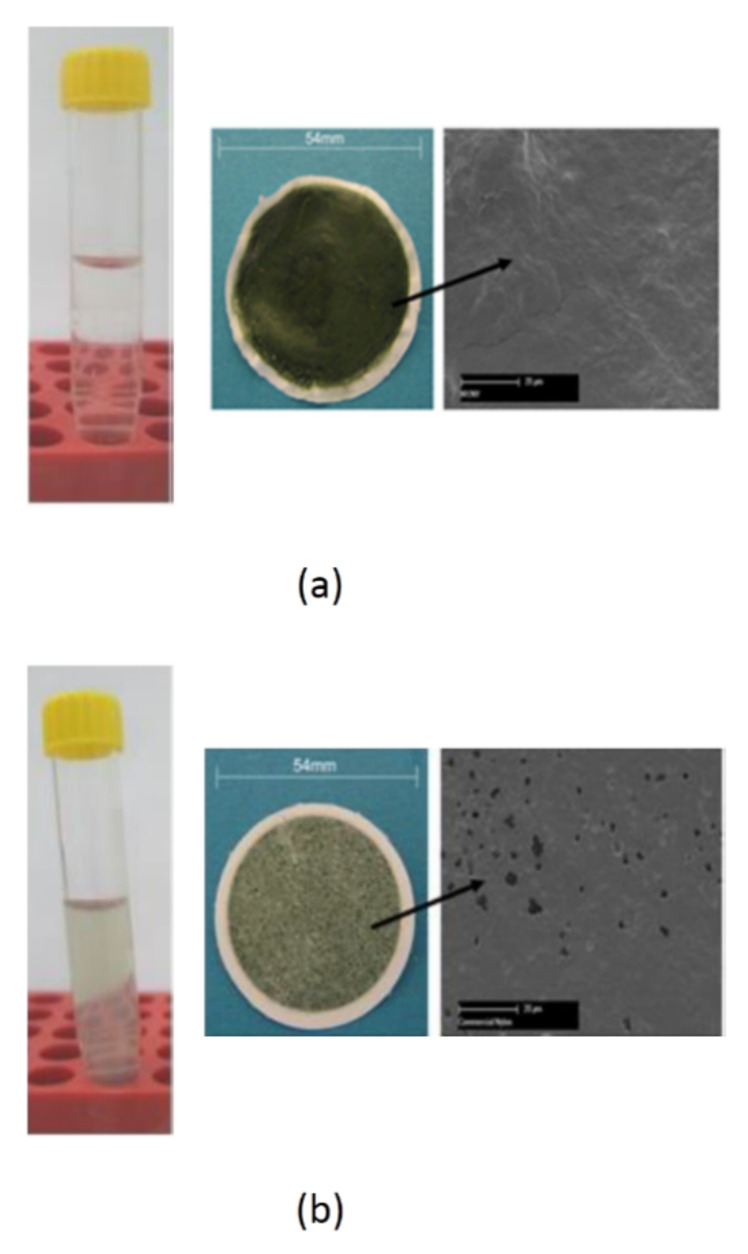
SEM images of the typical membranes after filtration and photographs of permeate using (**a**) an electrospun Nylon 6 membrane and (**b**) commercial Nylon membrane, respectively. Figure is adapted and modified from [105].

Apart from air filtration, the use of electrospun nanofiber membranes as a filter media for water filtration has been reported by a number of studies. The removal of contaminants in textile effluent using electrospun Nylon 6 membrane was investigated by Affandi and Razak (2017). The authors observed that the electrospun Nylon 6 membrane exhibited higher removal of suspended solids with approximately 99% as compared to the commercial Nylon membrane. The electrospun Nylon 6 membrane showed a clear permeate after the filtration (Figure 5a), whereas the commercial Nylon membrane gave a cloudy permeate after the filtration (Figure 5b) [105]. Another work published by Qureshi et al. (2017) reported on the performance of electrospun zein nanofiber as green and recyclable adsorbent for the removal of reactive black 5 (RB5) dye. The work described that the electrospun zein nanofibers exhibited exceptionally efficient performance in removing RB5 after contact within 20 min at room temperature and a normal working pH. The mechanism of dye–zein interaction was chiefly controlled via hydrophobic, electrostatic forces and hydrogen bond interactions [106]. Similar work by Bhaaumik et al. (2016) was also reported on the removal of RB5 using potential polyaniline nanofibers adsorbent. The adsorption results demonstrated that RB5 removal efficiency was slightly pH dependent and maximum removal was obtained at pH 2.0. The desorption experiments revealed that the PANI NFs can be reused effectively for five consecutive adsorption-desorption cycles without any loss of its original capacity [107].

Recent work studied on the adsorption performance of the nanofibrous sorbent on the removal of several oils (i.e., crude oil, silicone oil, gasoline, and diesel) and nonpolar organic solvents (i.e., toluene and *m*-xylene) was explored. The developed sorbent showed high sorption capacities, along with a rapid removal performance where the sorbent reached the equilibrium sorption capacity within a few minutes for oils and organic solvents. The feasibility of the designed hierarchically porous mat for oil spill removal was demonstrated by the treatment of real seawater and crude oil. The robustness and reusability of the sorbent were demonstrated through its regeneration by both mechanical recovery and toluene treatment [108]. It was recommended by Barroso-Solares et al. (2018) that the fabrication of electrospun fibrous mats with both hydrophobic and oleophilic properties might have contributed to the good performance as absorbents of oil from stable water in oil emulsions [109]. Jiang et al. (2015) investigated the use of iron oxide (Fe_3_O_4_) nanoparticles (NPs) in the fabrication of magnetic nanofibrous composite mat composed of polystyrene (PS)/polyvinylidene fluoride (PVDF) nanofibers. The mechanical property for both PS and PVDF nanofibrous sorbent was greatly improved and exhibited oleophilic and hydrophobic properties. The incorporation of magnetic Fe_3_O_4_ NPs in the composite mat helps in the easy recovery of the mats after the oil-in-water sorption process [110].

Gopal et al. (2006) investigated the solid-liquid separation using electrospun polyvinylidene fluoride (PVDF) nanofiber membranes. The fiber diameter of electrospun PVDF was approximately 380 nm, and the pore diameter was in a range of 4–10.6 µm. The authors found that the electrospun PVDF membranes were able to remove 5–10 µm particles without damaging the membrane’s structure or surface. Thus, these membranes have the potential to be used as a prefilter for ultrafiltration in order to minimize fouling formation [111]. In other study, Gopal et al. (2007) measured the solid-liquid separation of electrospun polysulfone (PS) membranes using the separation factor (S.F) (Equation (1)):

(1) $$S.F=\left(1-\frac{C_{permeate}}{C_{feed}}\right)\times 100\%.$$

where and are the concentrations of permeate solution and feed solutions, respectively [112].

The feasibility of electrospun membranes to remove heavy metal ions from aqueous solutions has been observed by several studies [113,114]. Mahapatra et al. [113] reported that the electrospun Fe_2_O_3_–Al_2_O_3_ nanocomposite fibers have successfully removed heavy metal ions from aqueous solutions. Other studies also reported that the electrospun polyvinyl chloride (PVC) nanofiber membranes not only able to remove 91% of cadmium ions, but also copper and lead ions with the removal efficiencies of 73% and 82%, respectively [114].

The water filtration properties of electrospun membrane were further enhanced by Nosang Myung and his team upon receiving a specific research grant from federal funding. The authors developed a nanofiber filter to improve the removal of nitrogen and phosphorus from contaminated water. The rapid growth of algae blooms as a result of the surplus of nitrogen and phosphorus in natural water has disturbed the aquatic ecosystem consequently causing the death of aquatic life. Meanwhile, the use of nutrient-polluted drinking water increased the severe health risks to humans, such as methemoglobinemia, and bladder cancer. However, the current approaches for nitrogen and phosphorus removal are not practicable for residents in rural communities. Reverse osmosis and conventional ion exchange are energy intensive with high capital, operational and maintenance costs. A water treatment method utilizing electrospun nanofibers uniquely suited for removing these nutrient pollutants and improving water quality control. The incorporation of additives has effectively removed the contaminants from groundwater in small, rural areas [115].

Recently, several improvements on the filtration performances of electrospun nanofiber membranes have been made such as membrane porosity, hydrophobicity, electrical conductivity and mechanical properties of the electrospun nanofiber membrane [116].

### 4.2. Defense and Protection Garment

The application of nanofibers in defense and protective garments is practically useful in order to increase the protection and survivability of people working in extreme conditions and hazardous situations. The requirements for such situations are to protect from injuries while also monitoring environmental hazards such as toxic gases. The improvement of fabric materials in performance and additional capabilities would be of huge assistance within professions such as the defense forces and emergency response services [117].

Recently, nanofibers with super strong, flexible fabrics that have ballistic properties with the embedment of carbon nanotubes have by now been fabricated. This new nanofiber is potential to be woven into fabrics, thus turning body armor suit to be as light as a cotton shirt, but bulletproof since carbon nanotubes are light and flexible, but enormously strong. This new nanofiber reinforced with carbon nanotubes has been reported to have properties of four times tougher than spider silk, and 17 times tougher than Kevlar, now used to make bulletproof vests commercially [118]. The research in this field polyvinylidene was then has been continued by Baniasadi et al. (2015) by spun nanofibers from polyvinylidene fluoride (PVDF) and its copolymer, polyvinvylidene fluoride trifluoroethylene (PVDF-TrFE). The fabricated nanofibers then were twisted into yarns, and then continued to twist the material into coils, similar to the same basic process used in making the conventional cable. The mechanical properties of the yarn and coils for the determination of their stretching ability and possible energy to be absorbed before the structural failure showed that the nanofibrous yarns achieved remarkable energy to failure of up to 98 J/g. Whereby, through an over twisting process, the fabricated polymeric coils out of twisted yarns stretched up to ~740% strain, indicated that the twisting process not only increases the failure strain but also increases overall strength and toughness. [119].

**Figure 6:**
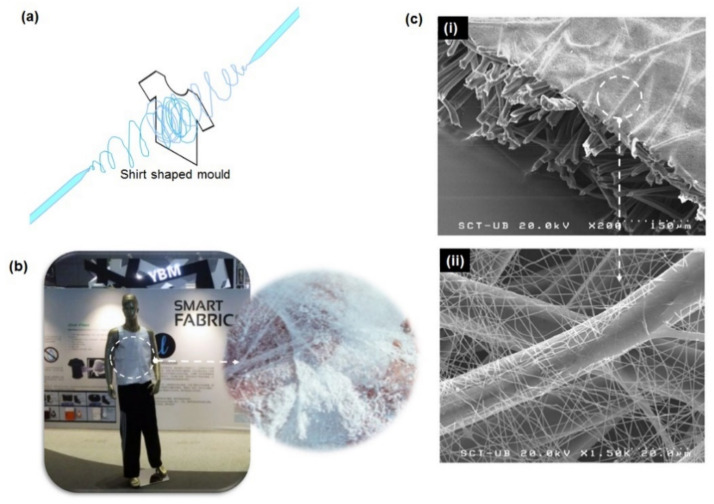
(**a**) Illustration of the molding technique for direct electrospinning. (**b**) The fabric from the Electroloom. (**c**) SEM micrographs of spunbonded polyamide (PA) 6 electrospun-viscose nonwoven membranes. Figures are adapted and modified from [122,123].

The following works done by other researchers have made an attempt in producing several nanofibers with the incorporation of functional materials into the electrospun nanofibers. One of the major focuses is to design material that can protect against toxic agents, both biological and chemical, by adding protective compounds to the polymer. Findings by Dhineshbabu et al. (2014) revealed that the incorporation of magnesium oxide (MgO) nanoparticles on Nylon 6 by an electrospinning process provide good flame resistance and antibacterial activity against Gram-negative E. coli and Gram-positive S. aureus pathogens. The antimicrobial effects of Nylon 6 fabrics for S. aureus are better than those for E. coli. The enhanced flame resistance, an increase in antibacterial activity, and an appropriate physical property led to a choice for protective clothing for soldiers [120]. Amongst the difficult things in the process of constructing fabrics from electrospinning is the making of self-made garments without any functional substrates. The progress research shows that a company known as Electroloom started the use of electrospinning for 3D printing custom for designing and fabricating wearable clothing [121]. This molding technique implicates the shaping mold of the desired clothing and electrospinning is used to construct thick layered electrospun nanofibers in a customized mold, as illustrated in Figure 6a. The constructed nanofibrous clothing is consequently removed from the mold and ready for wear (Figure 6b). As this technique remains a prototyping phase, certain complexity such as durability of the clothing, speed of fabrication and clothing design limitations should be addressed appropriately. Another limitation of this technique is it can only produce white fabric.

Fabrics produced from electrospun nanofibers membranes are usually in white, due to the translucency properties of polymers and light scattering phenomenon. Therefore, it is preferable for fashion colors wearable including for the nanofiber-based fabric to enrich their visual varieties. A research study reported by Yan et al. (2016) showed their good finding of using colored spinning solutions prior to electrospinning. The fabricated nanofibers membrane made of colored poly (vinyl butyral) (PVB)/cationic dye exhibited good color fastness. The decrease in the diameter of dyed fibers as the increase of dye concentration was also noticed, possibly affected by the increase of conductivity of the dye-polymer solution [124]. On top of that, the big concern for the engineered fabric made up of electrospun fibers is to retain their durability and functionalities as they are exposed to repeated washing and usage. The previous study reported the visible changes in the structural integrity of nanofibers through the formation of holes within a size of tens to hundreds of micrometers after 1500 cycles of repeated compression and torsion. Further additional use of resin dots has improved their structural integrity as nanofibers membrane. The simple hot press lamination of the nanofibers membrane onto the substrate revealed the least damage (Figure 6c) [123]. It is suggested that to keep the stress concentration at the bonded point of underlying fibers as low as possible. A single military laundering cycle with applied detergent on nanofibers and a triple layer of nanofiber/spunbond-laminated-nanofiber/spunbond demonstrated a reduction in aerosol retention efficiency to 66% and 86% of initial efficiency respectively [125]. Although studies on electrospun nanofibers are well established, the commercialization aspect of nanofibers in clothing is still in its infancy. Therefore, a scalable manufacturing process and commercialization of the nanofiber-based clothing should be highlighted in the future.

### 4.3. Medical Dressing

The use of electrospun nanofibers in the application of medical dressing has been of huge interest since their structure morphology resembles the native tissue structure. The natural polymer of chitin and chitosan are amazing materials that have multiple advantages to be used in medical dressing applications [126]. These polymer materials are both biocompatible and biodegradable and are found in abundance in nature as a renewable source. The embedment of these materials into nanofibers has acquired extra advantage due to the huge surface area which imparts on them additional properties especially in the biomedical fields. However, this task is not a simple one as chitin and chitosan are hard to electrospun due to the high molecular weights and the stiff back bone making the polymer solution very viscous for the electric field to stretch. Researchers found that the use of concentrated acetic acid as a solvent (90%) was able to reduce surface tension, besides the combination of solvents such as 1,1,1,3,3,3-hexafluoro-2-propanol (HFIP), trifluoroacetic acid (TFA) and dichloromethane (DCM) to be used in the electrospinning of chitosan-based nanofibers. Crosslinked nanofibers using glutaraldehyde vapors upon electrospinning helps to maintain their fibrous structure when immersed into aqueous media. The second strategy for producing smooth and nice nanofibers was by using blends of chitin or chitosan with other polymers such as PVA or PEO [127,128].

Another method to enhance the properties of the chiton nanofibers is plasma treatment. Annur et al. (2015) preloaded silver nitrate in the chitosan nanofibers via argon plasma treatment. This solid-state synthetic approach produced surface-immobilized silver nanoparticles in the absence of additional chemical reagents and also facilitated maximum exposure on the nanofiber surfaces. TEM examination confirmed that plasma-synthesized silver nanoparticles with an average diameter of 1.5 nm were mostly positioned on the nanofiber surfaces. Antibacterial performance assays based on bacterial inhibition zone measurements indicated a strong correspondence with the plasma dosages and the resulting surface-immobilized silver nanoparticles [129].

One of past research has conducted the fabrication of conductive scaffold from composite nanofibers of PANI/gelatin to promote the proliferation of the myoblast cells Li et al. (2006). Gelatin is often used in cardiac tissue engineering as it shares an almost similar molecular structure to collagen [130,131]. The 3% content of gelatin has significantly changed the physicochemical characteristics of the nanofibers composite. Further increment of PANI to gelatin volume ratio resulted in a decrease in fiber diameter of composite nanofibers. Different orientations of myoblast cell attachment were subjected to the concentration of PANI. It is found that the growth of cultured cells is more even on the smaller diameter of composite nanofibers as compared to the larger diameter nanofibers substrates. The research work by Li et al. (2006) [130] was further complemented by Mobarakeh (2011). It is mentioned that the surface modification and functionalization of PANI with biomolecules or dopants have allowed them to modify them with biological sensing elements, and exhibit different signaling pathways required for cellular processes. The use of conductive electrospun nanofibers provides a window of opportunity for the fabrication of highly selective, biocompatible, specific and stable nanocomposite scaffolds for organ-specific regeneration as it signifies the enhancement in cell proliferation and differentiation [132]. On the other hand, the antibacterial activity of honey/chitosan nanofibers incorporated with capsaicin and gold nanoparticles wound healing has been studied by Al-Musawi et al. (2020). The MTT assay demonstrated that the nanofibrous increased new cell proliferation compared with the untreated control, thus indirectly enhancing the wound-closure rate [133]. Another interesting portable electrospinning device operated by a battery has been developed by Liu et al. (2020). The function of this practical device is to produce copper monosulfide (CuS) composite nanofibers, which can be directly deposited onto the wound area, to simultaneously achieve rapid hemostasis and bacteria activity is ablated. It is reported that the healing time has been shortened to 18 days as compared to 26 days for the control sample [134]. Several similar studies have been conducted to increase the device functions such as reducing size for better practicality for outdoor use and also to develop new formulations utilizing different materials for other wound care treatment [135,136,137,138].

Recently, electrospun based products seem to be shifting from research to commercialization. Stellenbosch Nanofiber Company (SNC) Ltd. operated in Cape Town, South Africa has become as one of the platforms towards the commercialize nanofibers materials for the biomedical industry specialized for wound dressings, tissue engineering scaffolds, drug release materials and cell culture scaffolds. With support from InnovUS, the Stellenbosch University’s technology transfer company, a spin-out company was established to focus on the commercialization of the patented nanofiber production and processing technologies. The Stellenbosch Nanofiber Company managed to secure funding from private and institutional investors, and was officially launched in November 2011 [139]. In accordance with the recent study on the cell response to sterilized electrospun poly(E-caprolactone) scaffolds to aid tendon regeneration in vivo using PCL nanofibers manufactured from SNC Ltd., the sterilized electrospun PCL scaffold was found to function similarly to the gold standard autograft control in terms of cell response over a 6-week time period. Neither ethanol nor gamma sterilization had an observable effect on the functionality of the scaffold when compared to the autograft control. The manufacture of the electrospun PCL fibers did not have an effect on the performance of the scaffold, which would allow the manufacturing process to be easily outsourced and scaled up for commercial translation. Further long-term in vivo studies are required for electrospun PCL scaffold to become an alternative intervention for patients requiring tendon repair [140].

### 4.4. Home Furnishing

Another application for nanofibers which also in current development is home finishing products. These nanofibers materials are of particular interest in the area of home furnishing products, such as mattresses, bedding, and covered furniture. The potential benefits to consumers from the use of nanotechnology in textiles include fabrics with desirable enhancements, such as stain and water resistance, antistatic properties, moisture wicking, wrinkle resistance, and antibacterial properties. Textile end products, including clothing and home furnishings, are in intimate contact with consumers during every moment of their daily lives. The electrospun nanofiber-based products are expected to be patented and entered the market using nanotechnology by synergistic effects of nanodispersible fillers [141].

The previous research conducted by Knoff et al. (2008), has patented their innovative an allergen-barrier nanofibers fabric for mattress production purposes. A mattress which is having a microporous covering material comprising of a nanofibers layer comprising at least one porous layer of polymeric nanofibers having a number average diameter between about 50 nm to about 1000 nm, with the pore size of 0.01 to 10 um, a basis weight of 1 g/m^2^ and about 30 g/m^2^, and a Frazier air permeability of at least about 1.5 m^3^/min/m^2^ has been produced. Besides, they are also highlighted that the polymeric nanofibers can be adhered to one or more other fabrics to form an allergen-barrier fabric, for use in coverings such as pillow covers, mattress or pillow ticking, mattress pads, duvet covers and even linings for apparel [142].

Another interesting application of electrospun nanofibers is by incorporating these materials as a lightweight, yet dense, sound absorption alternative. Pollen Group Limited Company has integrated noise reduction products incorporating nanofibers, known as Phonix™ nanofibers, which are scientifically proven to help resolve the issues of noise in open plan spaces. The nanofibers convert sound energy to heat energy which is then dissipated. This nanofibers material has dulled the sound of speech to a more manageable and less distracting level [143].

On the other hand, a nanofibers pillow lining known as NanoDream™ were fabricated from medically approved, nonhazardous polymers, infused with the goodness of manuka extract to ward off bacteria and allergens is the marketable product of Revolution Fibres Limited, a New Zealand based company. The NanoDream electrospun nanofiber layer is so dense it creates a natural barrier to dust mites through size exclusion, as compared to the standard pillowcase and microfiber fabric [144]. Findings from this review indicate the electrospun nanofibers have been employed widely in home furnishing.

### 4.5. Food Packaging

The application of electrospun nanofibers in functional food packaging has become an evolving fascinating area to exploit and develop research for strength and barrier enhancements compared to conventional packaging materials [79,145]. According to the FDA, food packaging substances derived from electrospun nanofibers materials must possess biocompatibility and nontoxic properties. The developed research is meant for improving antimicrobial activity, rapid sensing and detection on the signaling microbiological and biochemical changes, in order to extend food shelf lives for long-term freshness.

**Figure 7:**
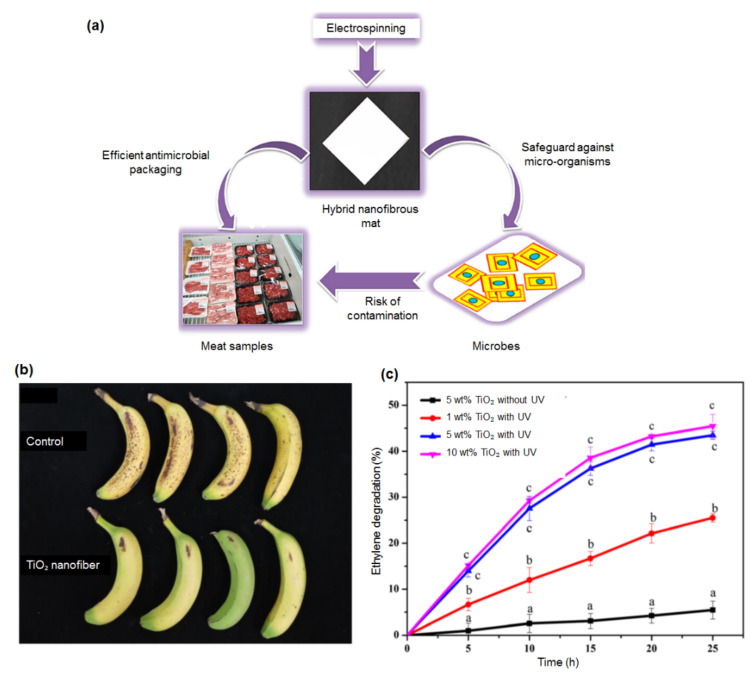
(**a**) Packaging material made of electrospun hybrid mats targeting for meat and meat-based products. (**b**) Photographs of bananas stored for 10 days covered with PP film (control) and nanofibers containing 5 wt% TiO_2_. (**c**) Ethylene photodegradation activity of a TiO_2_/PP nanofiber-based film. Figures are adapted and modified from [146,147].

The innovative food packaging composed of biodegradable polyurethane (PU) with antimicrobial properties targeted for meat and meat-products has been reported by the first time by Amna et al. (2014). Instead of mixing antimicrobial compounds directly with food, the incorporation of virgin olive oil and zinc oxide via electrospinning in packaging materials inhibits microbial activity at food surfaces where it is localized. The antibacterial activity was tested against two common foodborne pathogens such as Staphylococcus aureus and Salmonella typhimurium. The results revealed the functional additives of zinc oxide and olive oil, which are possessed with various antibacterial ingredients, flavonoids and antioxidants have improved the antimicrobial characteristic of PU nanofibers. Even in low concentration, ZnO embedded nanofiber-based film is potent enough to control the microbial growth due to the bacterial inhibitory properties of packaging material. Thus, the fabricated composite based packaging is able to inhibit food alteration and prevent spoilage of meat/meat-products (Figure 7a) [146].

Another profound study by Zhu et al. (2018) with the aim of producing a nanofiber-based film to enhance the photocatalytic activity of ethylene degradation and deceleration the ripening of banana has been demonstrated [147]. Their study reported that the polypropylene (PP) packaging made of nanofibers with 5wt% TiO_2_ exhibited the best nanoparticle uniformity and optimal ethylene photo-degradation activity as compared to the control sample (Figure 7b,c). This study is in line with other previous studies that described an increase in ethylene decomposition with increasing TiO_2_ concentration due to larger amounts of hydroxyl radicals generated by the TiO_2_ particles [148,149,150]. The removal of ethylene from the environment surrounding has resulted in prolonging bananas’ shelf lives, thus reducing the postharvest losses.

Apart from nanoparticles, a number of works on the use of antibacterial substances have been studied. Vega-Lugo et al. (2009) fabricated electrospun fibers from soy protein isolate (SPI) in poly (ethylene oxide) (PEO) and poly (lactic acid) (PLA) for the controlled release of a naturally occurring antimicrobial compound, allyl isothiocyanate (AITC). AITC is mostly found in mustard oil, and is effective against cheese-related fungi. Through this work, it is found that the concentration of AITC has significantly affected the fiber morphology, whereas it is reported that the AITC release was negligible under dry conditions but increased drastically as the relative humidity increased. It can be concluded that the AITC release is directly triggered by any change in the relative humidity. Aside from SPI and AITC, the application of cinnamon essential oil (CEO) embedded electrospun nanofibers also has been tested. According to Wen et al. (2016), the PVA nanofibrous packaging film containing CEO exhibited excellent antimicrobial activity against both *Staphylococcus aureus* and *Escherichia coli.* It is reported that the control (unpacked strawberry) had started bruises and decayed on the 4^th^ day. The strawberries packed with PVA/CEO/β-CD nanofibrous film showed no sign of decay even on the 6^th^ day. The use of PVA/CEO/β-CD nanofibrous film has practically prolonged the shelf-life of packed strawberries by preventing water losses and delaying the degradation of the cell wall, thus, holding the firmness of the packed fruits during storage [151,152,153]. The interactive behavior of the nanofibers with natural antimicrobials in active packaging applications may be an innovative idea to increase the preservation of cheese related foods [154].

### 4.6. Cosmetics

Electrospinning has the potential for use in various cosmetic applications for example facial masks, perfumes, deodorants and antiperspirants. A common feature of these applications is that they can be used in the form of a membrane which can be easily fabricated by electrospinning. The conceivable of incorporating active ingredients into electrospun nanofibers also makes this spinning process feasible for cosmetic applications. Yet, the application in the cosmetics product from nanofibers textiles material is relatively new. A reported study conducted by Fathi-Azarbayjani et al. (2010) indicated that electrospun fiber materials are influential to replace the conventional materials used in the current cosmetic facial masks. Their work demonstrated an anti-wrinkle nanofiber face mask incorporating collagen, novel vitamin and gold nanoparticles, with a control polymer degradation to discharge the active ingredients once contact with premoistened skin. The study reported that the high surface area to-volume ratio of the nanofiber mask will ensure maximum contact with the skin surface and help to enhance the skin permeation to restore its healthy appearance. The dry nature of face masks increases both the structural stability and their shelf lives when compared to premoistened cotton masks available in the market [155].

Another research study that utilized emulsion electrospinning to fabricate membranes containing volatile fragrance through poly(vinyl alcohol) (PVA) fibrous matrix to release encapsulated (R)-(+)-limonene for 15 days under ambient temperatures has been demonstrated [156]. The release profile of the fragrance from the electrospun nanofibers shows that this type of nanofibrous matrices with a high fragrance loading capacity is of great potential for applications in various fields, such as cosmetic with fragrance releasing, food packaging or textile items with fragrance release.

## 5. Global Nanofibers Industry

To shift the electrospun nanofibers from their prototyping to finished products is quite challenging. In contempt of the full potent ideas, electrospinning technology is facing several challenges in establishing its own position in the market and also substituting the current commercialized technologies. Researchers have been intent on evolving these materials, fabricating nanofibers from electrospinning over the past decade and transforming them into downstream and upstream products. The commercial usage of the electrospun nanofiber-based products is across various fields such as filtration and biomedical applications. Table 1 listed out several of the global nanofibers companies that already have commercialized their nanofiber-based products. These worldwide companies are among key players in the revolutionary creation of innovative electrospun nanofiber-based products.

### 5.1. Nanofibers in Global Business

The first technology enabling the production of nanofibers appeared on the global market in the 1980s. Donaldson, one of the leading companies in nanofiber-based applications brought out the nanofibers first time for advanced commercial applications namely as air filtration technology–Ultra-Web^®^ in 1981. The nanofibers presence did show a promise in reducing operating costs and improved efficiency as reported by Balamurugan et al. (2011) [157]. Until today, nanofibers technology has benefited numerous industries including filtration, military garment, cosmetics and others. The full potential of nanofibers technology has been tried to be realized in commercial consumer products. Recent developments in nanofibers production technology have opened up the possibilities of applying nanofibers along with various process improvements. In 2005, the company Elmarco launched the Nanospider^TM^, the recent technology in the world that enables nanofiber production for performance apparel on an industrial scale [157]. Since then, major nanofibers innovations and inventions from research laboratories are in the lead for commercialization to play a crucial part in the industrial economy. These nanofibers companies are engaged in new product developments, innovation, agreements, collaborations, mergers and acquisitions and strategic business activities to strengthen their market and increase distribution networks globally. This industry is expected to be highly competitive over the forecast period on account of the incorporation of new processes and product developments.

### 5.2. Market Trends of Electrospun Nanofibers

The rising popularity of an advanced product made of electrospun nanofiber-based materials is the primary factors that drive the growth of this market. Currently, the global nanofibers market is valued at USD 927 million in 2018 and is estimated to hit USD 4.3 billion by 2023 with an expected compound annual growth rate (CAGR) of 36.2% during the forecast period. The global market report done by PR Newswire is further analyzed by the following projected end-use application segments such as in packaging, automotive, electronics and semiconductors, aerospace, coatings and energy in segmented leading countries such as USA, Canada, China, Japan, Europe, North America and Asia regions. A research report by Global Industry Analysts Inc. entitled “Nanofibers: A Global Strategic Business Report” also has provided comprehensive reviews of the nanofibers market trends. As stated by the report, Asia–Pacific is the largest and the fastest growing market worldwide over the analysis period was conducted as a result of the strong public and private investments in nanotechnology. This is attributed to the high demand for nanofibers in performance apparel applications whereby their unique properties of high surface area, small fiber diameter, filtration properties, layer thinness, high permeability, and low basis weight, contribute to an outstanding product functionality [125]. Given by all this report, it has been presenting a current analysis on the nanofibers business as associated from an economic and industrial point of view and the expected key development in the future. The evaluation of the current status from a global standpoint, and information of the existing companies and suppliers related to these businesses are needed as this technology expands. Besides, the technical insights into nanofiber manufacturing by providing a review of materials, their specific applications and production methods are valuable to justify the importance of nanofibers market demand. In addition, it is also necessary to recognize current technical trends by providing a summary of the leading global R&D activities related to nanofibers, such as the issuance of patents related to nanofibers products, and the latest technological advancement. This information is beneficial to assist researchers in identifying opportunities for process and productivity enhancements, to strengthen the future of the long-run market growth, and subsequently provide considerable growth opportunities for nanofibers manufacturers globally [158].

## 6. Conclusions

The review presented here collectively highlighted the development of the scaling-up method in electrospinning technologies, current progress on the manufacturing scalability of electrospun nanofibers materials and the commercialization of electrospun nanofiber-based products. We started with a brief opening related to the history of electrospinning, followed by a detailed literature review of its genesis principle and typical apparatus. The discussion narrowed into their revolution as a potent technology for the production of nanoscale dimension materials with diversified compositions, and their properties and applications were revealed. The potential for the commercialization applications of electrospun nanofiber-based materials opened up a new avenue towards economically extending and enlightening the values of electrospinning products. Through electrospinning nanotechnology, ultra-strong, durable and specific function-oriented nanofiber-based fabrics have been now tested for a number of consumer product applications, such as filtration, defense and protection garments, medical dressings, home furnishings, food packaging and, also, in cosmetics. As mentioned, nanotechnology has overcome the limitations of applying conventional methods to impart certain properties to electrospun nanofiber-based materials. Electrospinning techniques have facilitated the fabrication of nonwoven fibers in nano-sized scale dimensions, which now has become possible for highly effective modern performance products. Thus, there is no doubt that, in the next incoming years, nanotechnology through electrospinning will penetrate every area of the functional textiles industry for more innovative product applications.

Yet, there are other concerns about the electrospinning technology that need to be addressed by the manufacturer, as well as the researchers. The issues are the hazardous vapors emitting from electrospinning solutions while forming nanofiber webs that need to be recovered or disposed of in an environmentally friendly manner. This will definitely involve additional equipment and cost. Another issue is the raised concern over possible health hazards to humans due to the inhalation of fibers, which needs to be taken into another, future study. From the authors’ point of view, the functionality of electrospun nanofibers has been greatly improved and proven to have abilities in various fields of application. However, the shelf-life issue of electrospun nanofibers fabrics prior to laundering and restoration is certainly limited, and insufficient work has been reported. The fabric should have both appropriate mechanical properties and the durability to withstand the physical and chemical stresses of laundering. The importance of the lifespan of electrospun nanofibers products should be thoroughly evaluated before actual industrial production begins. Finally, this review article offered the readers with interesting perspectives regarding applied products of electrospun nanofibers. We strongly believe that all the difficulties and constraints faced in the manufacturing, research and development, as well as processing, of electrospun nanofibers will be clearly resolved throughout advanced research exploration in the coming future.

## Figures and Tables

**Table 1:** Commercial electrospun nanofiber-applied products.

Product	Company	Application	Description	Company Website
3D Insert™	3D Biotek	Cell culture device for laboratory tissue culture	3D cell culture insert made of PS, PCL and PDLLGA	http://www.3dbiotekstore.com/ (accessed on 17 June 2021)
Absorv™	Zeus	Nonwoven fibrous materials	Bioabsorbable polymers made for of a wide variety of medical products for both preventive care and disease treatment	https://www.zeusinc.com (accessed on 17 June 2021)
Aeos™	Zeus	Nonwoven fibrous materials	Composed of a number of solid nodes interconnected by a matrix of thin fibrils, which allows to excel in such diverse applications as sutures	https://www.zeusinc.com (accessed on 17 June 2021)
Antidust half-mask	Esfil Techno	Filtering materials, half-masks, analytical tapes	Produce and develop highly efficient nonwoven polymer filtering materials made of micro and nano fibres	https://www.esfiltehno.ee/en/ (accessed on 17 June 2021)
AVflo™ Vascular Access Graft	Nicast Ltd.	Implantable medical devices	Is a multi-layered self-sealing vascular access graft made of electrospun polycarbonate urethane nanofibers	http://www.nicast.com/ (accessed on 17 June 2021)
Bio-Spun™	BioSurfaces Inc.	Novel three-dimensional (3D) nanofiber scaffolds	Electrospun fibers mimic natural extracellular matrix produced from variety of polymer materials and thicknesses	https://www.biosurfaces.us/ (accessed on 17 June 2021)
Bioweb™	Zeus	Nonwoven fibrous materials	Possesses a microporous nature that is similar to expanded PTFE (ePTFE) but achieves this without the nodes and fibrils associated with ePTFE	https://www.zeusinc.com (accessed on 17 June 2021)
BreaSAFE^®^	PARDAM NANO4FIBERS	Nanofiber based respirator	Provides an effective protection against microbes, particles, aerosols and odors and to a limited extent, also against toxic gases and vapours	https://www.nano4fibers.com/ (accessed on 17 June 2021)
Cytoweb^®^ Sheets	Espin Technologies	Cell culture device for laboratory tissue culture	Superior adhesion and proliferation of in vitro cell cultures, as compared to traditional lab plastic ware	http://www.espintechnologies.com/ (accessed on 17 June 2021)
Exceed^®^	Espin Technologies	Air filters media	A product designed to capture particulates while providing openings for un-hindered pathway for air flow	http://www.espintechnologies.com/ (accessed on 17 June 2021)
FERENA	Koken Ltd.	Nanofiber air filter	Innovative electrospinning technology targeted to expand a clean zone through nanofibrous filter membrane	https://www.koken-ltd.co.jp/ (accessed on 17 June 2021)
FiberTrap	FiberTrap	Bedding microfiber trapping material	The Fibertrap microfiber is patent-protected and manufactured with safe, green, recycled polymer materials through electrospinning, with a fluffy three-dimensional structure that immediately entangles the bed bugs upon contact	https://fibertrap.com/ (accessed on 17 June 2021)
Filter half-masks	Sorbent	Respiratory personal protective equipment (RPE)	Nanofibers filter half masks for respiratory personal protective equipment (RPE)	http://en.sorbent.su/ (accessed on 17 June 2021)
Filter NanoFiber	Astral Pool	Cartridge water filter	Self-cleaning cartridge filter which is able to filter particles of between 5 and 8 microns	https://www.astralpool.com/en/ (accessed on 17 June 2021)
FilterLayr	NanoLayr	Air filtration media	A product made of electrospun nanofiber which can be infused with active additives designed to trap and neutralize even the smallest airborne particles for filters and masks	https://www.nanolayr.com/ (accessed on 17 June 2021)
HealSmart™	PolyRemedy	Wound Dressings	Wound dressing with advanced technology with personalized therapeutics to improve wound healing outcomes	https://www.polyremedy.com/ (accessed on 17 June 2021)
Microfiber separation media	Nanopareil LLC	Microfiber separation media	Nanofiber based products that provide an enormous amount of specific surface area along with a controlled porosity and offers modular, off-the- shelf installation into existing manufacturing processes	https://www.nanopareil.com/ (accessed on 17 June 2021)
Micrograde NF filter	MANN+HUMMEL	Nanofiber-coated air filters	A filtration media that consists of a cellulose carrier material coated with extremely thin layers of ultra-fine polymer fibers	https://www.mann-hummel.com/en.html (accessed on 17 June 2021)
Mimetix^®^ scaffold	Electrospinning Company	Nanofibrous biomaterials for use in tissue-regenerative devices	Develops and manufactures variety of electrospun polymer scaffolds made from PLA, PLGA, PCL, PLCL and PAN	https://www.electrospinning.co.uk/ (accessed on 17 June 2021)
Naked filter	Liquidity Corporation	Nanofiber filter membranes	Nanofiber filter membranes fitted encapsulated drinking water purification cartridges	https://liquico.com/ (accessed on 17 June 2021)
Nanodream	NanoLayr	Nanofiber pillow lining	Anti-allergy bedding made of nonwoven electrospun nanofibers	https://www.nanolayr.com/ (accessed on 17 June 2021)
Nanofiber Solutions™	Nanofiber Solutions	Novel three-dimensional (3D) nanofiber scaffolds	Provide an ideal 3D substrate uses of aligned (NanoAligned™) or randomly oriented (NanoECM™ ) polymer nanofibers integrated into standard multi-well cell culture dishes	https://nanofibersolutions.com/ (accessed on 17 June 2021)
Nanofibrous biomaterials	NanoSpun Technologies	Nanofibrous biomaterials for use in tissue-regenerative devices	Develops and produces disruptive first-of-its-kind live-active biological tissues for cosmetics, well-being and health applications	http://www.nanospuntech.com/ (accessed on 17 June 2021)
Nanotrap™	Coway	Nanofibers Filter System	Water filter system which is effectively reduces bio-fouling materials such as cell debris, bacteria, viruses, with a patented electrospun coating	https://www.coway.com.my/ (accessed on 17 June 2021)
NeoDura™	Medprin Biotech GmbH	Dural Patch	Absorbable Dural repair patch made of degradable material poly-L-lactic acid and gelatin	http://www.medprin.com/ (accessed on 17 June 2021)
Neotherix scaffold	Neotherix	Bioresorbable scaffolds	Scaffolds possess a nonwoven three-dimensional architecture, comprising nano/micro-scale synthetic bioresorbable polymer fibres, which is highly porous scaffold structure supports the migration and proliferation of fibroblast cells from surrounding healthy skin tissue in order to facilitate healing of the wound	http://www.neotherix.com/ (accessed on 17 June 2021)
Nexture^®^	Lime	Technical textiles	Multi-functioning clothing with nanofiber membrane laminations which offer water and wind protection with a truly breathable difference, that can be worn during commutes, light workouts and various outdoor activities	http://limenano.com/ (accessed on 17 June 2021)
NnF CERAM^®^	PARDAM NANO4FIBERS	Inorganic nanofibers	Inorganic nanofibers are special function materials in the form of thin fibers, 3D cotton like ceramic, metal and powder like materials	https://www.nano4fibers.com/ (accessed on 17 June 2021)
NnF MBRANE^®^	PARDAM NANO4FIBERS	Filtration materials	A product suitable as separation membranes for many different products or filtration materials for water and air purification with very low pressure drop and very high filtration efficiency	https://www.nano4fibers.com/ (accessed on 17 June 2021)
Nonwoven nanofibers	Soft Materials and Technologies S.r.l.	Nonwoven nanofibers fibrous tissues, either in the form of nonwoven mats or as uniaxial aligned fibers	Provide nanostructured materials and nanofibers made by thermoplastic polymers, biodegradable polymers, optically active organic compounds and organic/inorganic hybrids	http://www.smtnano.com/ (accessed on 17 June 2021)
PK Papyrus^®^ covered coronary stent system	BIOTRONIK	Covered stent	Electrospun PU covered stent for use in the emergency treatment of acute coronary perforations	http://www.biotronik.com/ (accessed on 17 June 2021)
ProTura^®^ Nanofiber	United Air Specialists, Inc.	Cartridge filter	A cellulose nanofiber-based filtration media for cartridge elements for use in cartridge style dust collectors	http://www.clarcorindustrialair.com (accessed on 17 June 2021)
ReDura™	Medprin Biotech GmbH	Synthetic Dural Substitute	Fully degradable and absorbable, leaving no foreign body in-situ and is replaced by regenerated *Dura* tissue	http://www.medprin.com/ (accessed on 17 June 2021)
ResQFoam^TM^	Arsenal Medical	Therapeutic foams	In-situ forming polymeric foam made up of core-shell fibers for intracavity hemorrhage treatment	http://www.arsenalmedical.com/ (accessed on 17 June 2021)
Retissue™	Medprin Biotech GmbH	Bioresorbable Membrane	A synthetic fibrous membrane made of polylactic acid (PLA) and gelatin	http://www.medprin.com/ (accessed on 17 June 2021)
Return focus pod	IQ Commercial.	Acoustic soundproofing	Acoustic soundproofing material which has a destination touchdown point of soft walls which absorb noise in offices and offers a privacy for individual focused work	http://www.iqcommercial.co.nz/ (accessed on 17 June 2021)
RIFTELEN^®^ N15	FILTREX and PARDAM NANO4FIBERS	Filtration media for food product	Nanofibrous filtration membrane is suitable for filtration of cooking oils as well as liqueurs, spirits, wine, beer, lemonades, fruit juices and others	https://riftelen.com/ (accessed on 17 June 2021)
Smart mask	NASK HK	Nanofiber face mask	Face mask with unique nanofiber of air purifying and bacteria killing mask	http://nask.hk/ (accessed on 17 June 2021)
SNC BEST™	Stellenbosch Nanofiber Company	Nonwoven fibrous materials	Develop and manufacture nanofiber-based materials for medical applications	https://sncfibers.com/ (accessed on 17 June 2021)
SonoLayr	NanoLayr	Acoustic soundproofing	A lightweight sound absorption media, which is specifically designed to enhance the acoustic performance of any sound-control product	https://www.nanolayr.com/ (accessed on 17 June 2021)
SpinCare™	Nanomedic Technologies	Commercialized electrospinning wound treatment portable device	Utilizes proprietary Electrospun Healing Fiber (EHF™) technology to treat even the most severe and complicated wounds with a single application, eliminating the need for painful re-dressings	https://nanomedic.com/ (accessed on 17 June 2021)
SpurTex^®^	SPUR	Air filter materials	Materials with high air-filter performance when separating ultrafine particles	https://www.spur.cz/cs/ (accessed on 17 June 2021)
StypCel™	Medprin Biotech GmbH	Absorbable Hemostat	For treatment in hemostasis in capillary and venous, as well as small artery, bleeding	http://www.medprin.com/ (accessed on 17 June 2021)
Technoweb™	Lime	Nanofiber-based filters	Nanofiber filter medias that allow surface filtration to improve the efficiency and extend the lifetime of an HVAC and automotive engine and liquid and dust collectors	http://limenano.com/ (accessed on 17 June 2021)
Tubular and disc scaffold	SKE Research Equipment	Scaffold for 3D cell cultures and tissue engineering applications	Tubular and disc scaffolds made of silk fibroin and PCL	https://www.ske.it/ (accessed on 17 June 2021)
Ultra-Web^®^	Donaldson	Cartridge air filters	Cartridge air filters that offer a longer filter life, lower pressure drop and a reduction in energy usage	https://www.donaldson.com/en-sg/ (accessed on 17 June 2021)
Wetlaid Nonwoven Fabrics	Hirose Paper Mfg Co., Ltd.	Nanofiber-coated paper	Manufactures wetlaid nonwoven electrospun nanofibers fabrics	https://www.hirose-paper-mfg.co.jp/ (accessed on 17 June 2021)
XantuLayr^®^	NanoLayr	Fiber-reinforced composite	A product consists of interleaving nanofiber veil, for use in fiber reinforced thermoset polymer composite materials	https://www.nanolayr.com/ (accessed on 17 June 2021)

## Abbreviations

1D	One-dimensional
2D	Two-dimensional
3D	Three-dimensional
Ag	Silver
CNTs	Carbon nanotubes
NaBH_4_	Sodium borohydride
PAN	Polyacrylonitrile
PCL	Polycaprolactone
TiO_2_	Titanium dioxide
SiO_2_	Silicon dioxide
ZnO	Zinc oxide
PEO	Polyethylene oxide
UV	Ultraviolet
TE	Thermoelectric
RTILs	Room temperature ionic liquids
SVA	Solvent vapour annealing
PM	Particulate matter
Fe_2_O_3_-Al_2_O_3_	Iron oxide-aluminum oxide
PVC	Polyvinyl chloride
PVDF	Polyvinylidene fluoride
SF	Separation factor
PVDF-TrFE	Polyvinylidene fluoride-trifluoroethylene
MgO	Magnesium oxide
PA6	Polyamide 6
HFIP	Hexafluoroisopropanol
TFA	Trifluoroacetic acid
DCM	Dichloromethane
PANI	Polyaniline
PU	Polyurethane
SPI	Soy protein isolates
PLA	Polylactic acid
AITC	Allyl isothiocyanate
PP	Polypropylene
TEM	Transmission electron microscopy
CuS	Copper monosulfide

## References

[1] Patra J.K., Gouda S. (2013). Application of nanotechnology in textile engineering: An overview. J. Eng. Technol. Res..

[2] Gulrajani M.L., Gupta D. (2011). Emerging techniques for functional finishing of textiles. Ind. J. Fibre Text. Res..

[3] Som C., Gallen E.S. (2007). NanoTextiles: Functions, Nanoparticles and Commercial Applications. https://www.empa.ch/documents/56122/328606/NanoSafeTextiles_1.pdf/b2add656-265b-42df-9196-f2768d773748.

[4] Wong Y.W.H., Yuen C.W.M., Leung M.Y.S., Ku S.K.A., Lam H.L.I. (2006). Selected applications of nanotechnology in textiles. AUTEX Res. J..

[5] Raj S., Jose S., Sumod U.S., Sabitha M. (2012). Nanotechnology in cosmetics: Opportunities and challenges. J. Pharm. Bioallied Sci..

[6] Jebamalar Leavline E., Asir Antony Gnana Singh D., Prasannanayagi S., Kiruthika R. (2015). A compendium of nano materials and their applications in smart nano textiles. Res. J. Nanosci. Nanotechnol..

[7] Xin J.H., Daoud W.A., Kong Y.Y. (2004). A new approach to UV-blocking treatment for cotton fabrics. Text. Res. J..

[8] Yeo S.Y., Lee H.J., Jeong S.H. (2003). Preparation of nanocomposite fibers for permanent antibacterial effect. J. Mater. Sci..

[9] Afifi A.M., Nakano S., Yamane H., Kimura Y. (2010). Electrospinning of continuous aligning yarns with a ‘Funnel’ Target. Macromol. Mater. Eng..

[10] Pillai C.K.S., Sharma C.P. (2009). Electrospinning of chitin and chitosan nanofibres. Trends Biomater. Artif. Organs.

[11] De Vrieze S., De Clerck K. (2009). 80 years of electrospinning. International Conference on Latest Advances in High-Tech Textiles and Textile-Based Materials.

[12] Persano L., Camposeo A., Tekmen C., Pisignano D. (2013). Industrial upscaling of electrospinning and applications of polymer nanofibers: A review. Macromol. Mater. Eng..

[13] Luo C.J., Stoyanov S.D., Stride E., Pelan E., Edirisinghe M. (2012). Electrospinning versus fibre production methods: From specifics to technological. Chem. Soc. Rev..

[14] Nayak R., Padhye R., Kyratzis I.L., Truong Y.B., Arnold L. (2012). Recent advances in nanofibre fabrication techniques. Text. Res. J..

[15] Agarwal S., Wendorff J.H., Greiner A. (2008). Use of electrospinning technique for biomedical applications. Polymer.

[16] Yuan X., Zhang Y., Dong C., Sheng J. (2004). Morphology of ultrafine polysulfone fibers prepared by electrospinning. Polym. Int..

[17] Huang W., Zou T., Li S., Jing J., Xia X., Liu X. (2013). Drug-loaded zein nanofibers prepared using a modified coaxial electrospinning process. J. Am. Assoc. Pharm. Sci..

[18] Pillay V., Dott C., Choonara Y.E., Tyagi C., Tomar L., Kumar P., du Toit L.C., Ndesendo V.M. (2013). A review of the effect of processing variables on the fabrication of electrospun nanofibers for drug delivery applications. J. Nanomater..

[19] Garg K., Bowlin G.L. (2011). Electrospinning jets and nanofibrous structures. Biomicrofluidics.

[20] Ifegwu O.C., Anyakora C., Tański T.A., Jarka P., Matysiak W. (2018). The place of electrospinning in separation science and biomedical engineering. Electrospinning Method Used to Create Functional Nanocomposites Films.

[21] Lei T., Peng Q., Chen Q., Xiong J., Sun D. (2017). Alignment of electrospun fibers using the whipping instability. Mater. Lett..

[22] Xue J., Wu T., Dai Y., Xia Y. (2019). Electrospinning and electrospun nanofibers: Methods, materials, and applications. Chem. Rev..

[23] Cooper C.J., Mohanty A.K., Misra M. (2018). Electrospinning process and structure relationship of biobased poly (butylene succinate) for Nanoporous Fibers. ACS Omega.

[24] Qin X., Subianto S. (2017). Electrospun Nanofibers.

[25] Khan N. (2012). Applications of electrospun nanofibers in the biomedical field. SURG J..

[26] Luzio A., Canesi E.V., Bertarelli C., Caironi M. (2014). Electrospun polymer fibers for electronic applications. Materials.

[27] Subbiah T., Bhat G.S., Tock R.W., Parameswaran S., Ramkumar S.S. (2005). Electrospinning of nanofibers. J. Appl. Polym..

[28] Frenot A., Chronakis I.S. (2003). Polymer nanofibers assembled by electrospinning. Curr. Opin. Colloid Interface Sci..

[29] Park S., Park K., Yoon H., Son J., Min T., Kim G. (2007). Apparatus for preparing electrospun nanofibers: Designing an electrospinning process for nanofiber fabrication. Polym. Int..

[30] Ibrahim H.M., Klingner A. (2020). A review on electrospun polymeric nanofibers: Production parameters and potential applications. Polym. Test..

[31] Haider A., Haider S., Kang I.-K. (2018). A comprehensive review summarizing the effect of electrospinning parameters and potential applications of nanofibers in biomedical and biotechnology. Arab. J. Chem..

[32] Tan E.P.S., Lim C.T. (2006). Mechanical characterization of nanofibers: A review. Compos. Sci. Technol..

[33] Nisbet D.R., Forsythe J.S., Shen W., Finkelstein D.I., Horne M.K. (2009). A review of the cellular response on electrospun Nanofibers for tissue engineering. J. Biomater. Appl..

[34] Kanani A.G., Bahram. S.H. (2010). Review on electrospun nanofibers scaffold and biomedical applications. Trends Biomater. Artif. Organs.

[35] Fang J., Niu H., Lin T., Wang X. (2008). Applications of electrospun nanofibers. Chin. Sci. Bull..

[36] Thavasi V., Singh G., Ramakrishna S. (2008). Electrospun nanofibers in energy and environmental applications. Energy Environ. Sci..

[37] Rafiei S., Maghsoodloo S., Noroozi B., Mottaghitalab V., Haghi A.K. (2013). Mathematical modeling in electrospinning process of nanofibers: A detailed review. Cell. Chem. Technol..

[38] Angammana C.J., Jayaram S.H. (2011). Analysis of the effects of solution conductivity on electrospinning process and fiber morphology. IEEE Trans. Ind. Appl..

[39] Motamedi A.S., Mirzadeh H., Hajiesmaeilbaigi F., Bagheri-Khoulenjani S., Shokrgozar M. (2017). Effect of electrospinning parameters on morphological properties of PVDF nanofibrous scaffolds. Prog. Biomater..

[40] Henriques C., Vidinha R., Botequim D., Borges J.P., Silva J.A.M.C. (2009). A systematic study of solution and processing parameters on nanofiber morphology using a new electrospinning apparatus. J. Nanosci. Nanotechnol..

[41] Khanlou H.M., Sadollah A., Ang B.C., Kim J.H., Talebian S., Ghadimi A. (2014). Prediction and optimization of electrospinning parameters for polymethyl methacrylate nanofiber fabrication using response surface methodology and artificial neural networks. Neural Comput. Appl..

[42] Zhang S., Campagne C., Salaün F. (2019). Influence of solvent selection in the electrospraying process of polycaprolactone. App. Sci.

[43] Reneker D.H., Yarin A.L. (2008). Electrospinning jets and polymer nanofibers. Polymer.

[44] Zhang H. (2011). Effects of electrospinning parameters on morphology and diameter of electrospun PLGA/MWNTs fibers and cytocompatibility in vitro. J. Bioact. Compat. Polym..

[45] Elsabee M.Z., Naguib H.F., Morsi R.E. (2012). Chitosan based nanofibers, review. Mater. Sci. Eng. C.

[46] Fadil F., Affandi N.D.N., Misnon M.I. (2019). Mechanical behaviour of MWCNTs reinforced electrospun nanofibres. J. Macromol. Sci. Part A.

[47] Qiao B., Ding X., Hou X., Wu S. (2011). Study on the electrospun CNTs/polyacrylonitrile-based nanofiber composites. J. Nanomater.

[48] Jiang Q., Fu G., Xie D., Jiang S., Chen Z., Huang B., Zhao Y. (2012). Preparation of carbon nanotube/polyaniline nanofiber by electrospinning. Procedia Eng..

[49] Chakoli A.N., Wan J., Feng J.T., Amirian M., Sui J.H., Cai W. (2009). Functionalization of multiwalled carbon nanotubes for reinforcing of poly (l-lactide-co-ɛ-caprolactone) biodegradable copolymers. Appl. Surf. Sci..

[50] Nayak R., Kyratzis I.L., Truong Y.B., Padhye R., Arnold L. (2012). Melt-electrospinning of polypropylene with conductive additives. J. Mater. Sci..

[51] Teo W.E., Inai R., Ramakrishna S. (2011). Technological advances in electrospinning of nanofibers. Sci. Technol. Adv. Mater..

[52] Sahay R., Thavasi V., Ramakrishna S. (2011). Design modifications in electrospinning setup for advanced applications. J. Nanomater..

[53] Teo W.E., Ramakrishna S. (2006). A review on electrospinning design and nanofibre assemblies. Nanotechnology.

[54] Niu H., Lin T. (2012). Fiber generators in needleless electrospinning. J. Nanomater..

[55] Niu H., Wang X., Lin T. (2012). Needleless electrospinning: Influences of fibre generator geometry. J. Text. Ins..

[56] Niu H., Lin T., Wang X. (2009). Needleless electrospinning. I. A comparison of cylinder and disk nozzles. J. Appl. Polym. Sci..

[57] Niu H., Wang X., Lin T. (2012). Finite Element-aided electric field analysis of needleless electrospinning. Computational Finite Element Methods in Nanotechnology.

[58] Yalcinkaya F., Yalcinkaya B., Jirsak O., Haider S., Haider A. (2016). Dependent and Independent Parameters of Needleless Electrospinning. Electrospinning—Material, Techniques, and Biomedical Applications.

[59] IME Technologies Climate Controlled Electrospinning. https://www.ime-electrospinning.com/electrospinning-controlled-environmental/.

[60] Kim J.H., Lee J.H., Kim J.Y., Kim S. (2018). Synthesis of aligned TiO2 nanofibers using electrospinning. Appl. Sci..

[61] Ismaya E.P., Diantoro M., Kusumaatmaja A., Triyana K. (2016). Preparation of PVA/TiO_2_ composites nanofibers by using electrospinning method for photocatalytic degradation. IOP Conf. Ser..

[62] Chen Y., Shafiq M., Liu M., Morsi Y., Mo X. (2020). Advanced fabrication for electrospun three-dimensional nanofiber aerogels and scaffolds. Bioact. Mater..

[63] Chainani A., Hippensteel K.J., Kishan A., Garrigues N.W., Ruch D.S., Guilak F., Little D. (2013). Multilayered electrospun scaffolds for tendon tissue engineering. Tissue Eng. A.

[64] Sun B., Li J., Liu W., Aqeel B.M., El-Hamshary H., Al-Deyab S.S., Mo X. (2014). Fabrication and characterization of mineralized P(LLA-CL)/SF three-dimensional nanoyarn scaffolds. Iran. Polym. J..

[65] Kim T.G., Chung H.J., Park T.G. (2008). Macroporous and nanofibrous hyaluronic acid/collagen hybrid scaffold fabricated by concurrent electrospinning and deposition/leaching of salt particles. Acta Biomater..

[66] In K.S., Won H.S., Sang Y.L., Sang H.L., Seong J.H., Myung C.L., Lee S.J. (2009). Chitosan nano-/microfibrous double-layered membrane with rolled-up three-dimensional structures for chondrocyte cultivation. J. Biomed. Mater. Res..

[67] Jiang J., Mark A.C., Matthew J.T., Hongjun W., Matthew R.M., Jingwei X. (2015). Expanding two-dimensional electrospun nanofiber membranes in the third dimension by a modified gas-foaming technique. ACS Biomater. Sci. Eng..

[68] Ye K., Kuang H., You Z., Morsi Y., Mo X. (2019). Electrospun nanofibers for tissue engineering with drug loading and release. Pharmaceutics.

[69] Wu C., An Q., Li D., Wang J., He L., Huang C., Li Y., Zhu W., Mo X. (2014). A novel heparin loaded poly (l-lactide-co-caprolactone) covered stent for aneurysm therapy. Mater. Lett..

[70] Phan D.N., Dorjjugder N., Saito Y., Taguchi G., Ullah A., Kharaghani D., Kim I.S. (2020). The synthesis of sil-ver-nanoparticle-anchored electrospun polyacrylonitrile nanofibers and a comparison with as-spun silver/polyacrylonitrile nanocomposite membranes upon antibacterial activity. Polym. Bull..

[71] Caratão B., Carneiro E., Sá P., Almeida B., Carvalho S. (2014). Properties of electrospun TiO_2_ nanofibers. J. Nanotechnol..

[72] Zong X., Cai Y., Sun G., Zhao Y., Huang F., Song L., Hu Y., Fong H., Wei Q. (2015). Fabrication and characterization of electrospun SiO2 nanofibers absorbed with fatty acid eutectics for thermal energy storage/retrieval. Sol. Energy Mater. Sol. Cells.

[73] Miao F., Shao C., Li X., Wang K., Lu N., Liu Y. (2016). Electrospun carbon nanofibers/carbon nanotubes/polyaniline ternary composites with enhanced electrochemical performance for flexible solid-state supercapacitors. ACS Sustain. Chem. Eng..

[74] Di Mauro A., Zimbone M., Fragalà M.E., Impellizzeri G. (2016). Synthesis of ZnO nanofibers by the electrospinning process. Mater. Sci. Semicond. Process..

[75] Raeesi F., Nouri M., Haghi A.K. (2009). Electrospinning of polyaniline-polyacrylonitrile blend nanofibers. e-Polym..

[76] Schneider H.E., Steuber J.G., Du W., Mortazavi M., Bullock D.W. (2016). Polyethylene oxide nanofiber production by electrospinning. J. Ark. Acad. Sci..

[77] Pilehvar-Soltanahmadi Y., Akbarzadeh A., Moazzez-Lalaklo N., Zarghami N. (2016). An update on clinical applications of electrospun nanofibers for skin bioengineering. Artif. Cells Nanomed. Biotechnol..

[78] Zafar M., Najeeb S., Khurshid Z., Vazirzadeh M., Zohaib S., Najeeb B., Sefat F. (2016). Potential of electrospun nanofibers for biomedical and dental applications. Materials.

[79] Torres-Giner S., Lagaron. J.M. (2011). Electrospun nanofibers for food packaging applications. Multifunctional and Nanoreinforced Polymers for Food Packaging.

[80] Nejati-Koshki K., Pilehvar-Soltanahmadi Y., Alizadeh E., Ebrahimi-Kalan A., Mortazavi Y., Zarghami N. (2017). Development of Emu oil-loaded PCL/collagen bioactive nanofibers for proliferation and stemness preservation of human adipose-derived stem cells: Possible application in regenerative medicine. Drug Dev. Ind. Pharm..

[81] Qasim S.B., Zafar M.S., Najeeb S., Khurshid Z., Shah A.H., Husain S., Rehman I.U. (2018). Electrospinning of chitosan-based solutions for tissue engineering and regenerative medicine. Int. J. Mol. Sci..

[82] Xingxing Y., Linpeng F., Linlin M., Yunyi W., Si L., Fan Y., Xiaohan P., Gejie L., Dongdong Z., Hongsheng W. (2017). Green electrospun Manuka honey/silk fibroin fibrous matrices as potential wound dressing. Mater. Des..

[83] Moreta S., Cahyono E., Affandi N.D.N., Fadil F., Kurniawan C. (2020). Polymeric and non-polymeric nanofiber of cinnamaldehyde from cinnamon oil (Cinnamomum zeylanicum). J. Phys. Conf. Ser..

[84] Topuz F., Holtzl T., Szekely G. (2021). Scavenging organic micropollutants from water with nanofibrous hypercrosslinked cy-clodextrin membranes derived from green resources. Chem. Eng. J..

[85] Balzer C., Armstrong M., Shan B., Huang Y., Liu J., Mu B. (2018). Modeling nanoparticle dispersion in electrospun nanofibers. Langmuir.

[86] Hulsey S., Absar S., Choi H. (2018). Investigation of simultaneous ultrasonic processing of polymer-nanoparticle solutions for electrospinning of nanocomposite nanofibers. J. Manuf. Process..

[87] Hulsey S., Absar S., Choi H. (2017). Comparative study of polymer dissolution techniques for electrospinning. Procedia Manuf..

[88] Nieminen H.J., Laidmäe I., Salmi A., Rauhala T., Paulin T., Heinämäki J., Hæggström E. (2018). Ultrasound-enhanced electrospinning. Sci. Rep..

[89] Rouxel D., Hadji R., Vincent B., Fort Y. (2011). Effect of ultrasonication and dispersion stability on the cluster size of alumina nanoscale particles in aqueous solutions. Ultrason. Sonochem..

[90] Zhang C.L., Yu S.H. (2014). Nanoparticles meet electrospinning: Recent advances and future prospects. Chem. Soc. Rev..

[91] Datta R.S., Said S.M., Shahrir S.R., Abdullah N., Sabri M.F.M., Balamurugan S., Miyazaki Y., Hayashi K., Hashim N.A., Habiba U. (2015). Ionic liquid entrapment by an electrospun polymer nanofiber matrix as a high conductivity polymer electrolyte. RSC Adv..

[92] Matteson M.J., Orr C. (2017). Filtration: Principles and Practices.

[93] Heyang Y., Zhen H. (2015). Integrating membrane filtration into bioelectrochemical systems as next generation energy-efficient wastewater treatment technologies for water reclamation: A review. Bioresour. Technol..

[94] Russell S.J. (2007). Handbook of Nonwovens.

[95] Ruth O., Daphne H., Noemí M., Antonio G., Carlos N., Ángeles B. (2014). Application of multi-barrier membrane filtration technologies to reclaim municipal wastewater for industrial use. J. Sep. Purif. Rev..

[96] Mohammadzadehmoghadam S., Dong Y., Barbhuiya S., Guo L., Liu D., Umer R., Qi X., Tang Y. (2016). Electrospinning: Current status and future trends. Nano-Size Polym..

[97] Ultra-web Media Technology. https://www.donaldson.com/en-be/industrial-dust-fume-mist/technical-articles/ultra-web-media-technology/.

[98] Heikkila P., Taipale A., Lehtimaki M., Harlin A. (2008). Electrospinning of polyamides with different chain compositions for filtration application. Polym. Eng. Sci..

[99] Huang X., Jiao T., Liu Q., Zhang L., Zhou J., Li B., Peng Q. (2019). Hierarchical electrospun nanofibers treated by solvent vapor annealing as air filtration mat for high-efficiency PM2.5 capture. Sci. China Mater..

[100] Liu C., Hsu P.-C., Lee H.-W., Zheng G., Liu N., Li W., Cui Y. (2015). Transparent air filter for high-efficiency PM2.5 capture. Nat. Commun..

[101] Bien Y., Wang S., Zhang L., Chen C. (2020). Influence of fiber diameter, filter thickness, and packing density on PM2.5 removal efficiency of electrospun nanofiber air filters for indoor applications. Build. Environ..

[102] Molaeipour Y., Gharehaghaji A.A., Bahrami H. (2015). Filtration performance of cigarette filter tip containing nanofibrous filter. J. Ind. Text..

[103] Matulevicious J., Kliucininkas L., Prasauskas T., Buivydiene D., Martuzevicius D. (2016). The comparative study of a aerosol filtration byelectrospun polyamide, polyvinyl acetate, polyacrylonitrile and cellulose acetate nanofiber media. J. Aerosol Sci..

[104] Jaroszczyk T., Petrik S., Donahue K. (2009). Recent development in heavy duty engine air filtration and the role of nanofiber filter media. J. KONES.

[105] Affandi N.D.N., Razak N.N.A. (2017). Removal of pigment from textile wastewater by electrospun nanofibre membrane. J. Mech. Eng..

[106] Qureshi U.A., Khatri Z., Ahmed F., Khatri M., Kim I.-S. (2017). Electrospun zein nanofiber as a green and recyclable adsorbent for the removal of reactive black 5 from the aqueous phase. ACS Sustain. Chem. Eng..

[107] Bhaumik M., McCrindle R.I., Maity A., Agarwal S., Gupta V.K. (2016). Polyaniline nanofibers as highly effective re-usable adsorbent for removal of reactive black 5 from aqueous solutions. J. Colloid Interface Sci..

[108] Topuz F., Abdulhamid M.A., Nunes S.P., Szekely G. (2020). Hierarchically porous electrospun nanofibrous mats produced from intrinsically microporous fluorinated polyimide for the removal of oils and non-polar solvents. Environ. Sci. Nano.

[109] Barroso-Solares S., Pinto J., Nanni G., Fragouli D., Athanassiou A. (2018). Enhanced oil removal from water in oil stable emulsions using electrospun nanocomposite fiber mats. RSC Adv..

[110] Jiang Z., Tijing L.D., Amarjargal A., Park C.H., An K.-J., Shon H.K., Kim C.S. (2015). Removal of oil from water using magnetic bicomponent composite nanofibers fabricated by electrospinning. Compos. Part B Eng..

[111] Gopal R., Kaur S., Ma Z., Chan C., Ramakrishna S., Matsuura T. (2006). Electrospun nanofibrous filtration membrane. J. Membr. Sci..

[112] Gopal R., Kaur S., Feng C., Chan C., Ramakrishna S., Tabe S., Matsuura T. (2007). Electrospun nanofibrous polysulfone membranes as pre-filters: Particulate removal. J. Membr. Sci..

[113] Mahapatra A., Mishra B.G., Hota G. (2013). Electrospun Fe2O3–Al2O3 nanocomposite fibers as efficient adsorbent for removal of heavy metal ions from aqueous solution. J. Hazard. Mater..

[114] Sang Y., Li F., Gu Q., Liang C., Chen J. (2008). Heavy metal-contaminated groundwater treatment by a novel nanofiber membrane. Desalination.

[115] Peter K.T., Myung N.V., Cwiertny D.M. (2018). Surfactant-assisted fabrication of porous polymeric nanofibers with surface-enriched iron oxide nanoparticles: Composite filtration materials for removal of metal cations. Environ. Sci. Nano.

[116] Ahmed F.E., Lalia B.S., Hashaikeh R. (2015). A review on electrospinning for membrane fabrication: Challenges and applications. Desalination.

[117] Sinha M.K., Das B.R., Prasad N., Kishore B., Kumar K. (2018). Exploration of nanofibrous coated webs for chemical and biological protection. Zaštita Mater..

[118] Yao J., Bastiaansen C.W., Peijs T. (2014). High strength and high modulus electrospun nanofibers. Fibers.

[119] Baniasadi M., Huang J., Xu Z., Moreno S., Yang X., Chang J., Quevedo-Lopez M.A., Naraghi M., Minary-Jolandan M. (2015). High-performance coils and yarns of polymeric piezoelectric nanofibers. ACS Appl. Mater. Interfaces.

[120] Dhineshbabu N.R., Karunakaran G., Suriyaprabha R., Manivasakan P., Rajendran V. (2014). Electrospun MgO/Nylon 6 hybrid nanofibers for protective clothing. Nano-Micro Lett..

[121] White J., Foley M., Rowley A. (2015). A novel approach to 3D-printed fabrics and garments. 3D Print. Addit. Manuf..

[122] How Electroloom’s Clothes-Printing Revolution Died. https://www.engadget.com/2017–09–14-electroloom-clothes-printing-startup-death-aaron-rowley.html.

[123] Faccini M., Vaquero C., Amantia D. (2012). Development of protective clothing against nanoparticle based on electrospun nanofibers. J. Nanomater..

[124] Yan X., You M.H., Lou T., Yu M., Zhang J.C., Gong M.G., Lv F.Y., Huang Y.Y., Long Y.Z. (2016). Colorful hydrophobic poly (vinyl butyral)/cationic dye fibrous membranes via a colored solution electrospinning process. Nanoscale Res. Lett..

[125] Graham K., Gogins M., Schreuder-Gibson H. (2004). Incorporation of electrospun nanofibers into functional structures. Int. Nonwovens J..

[126] Liu H., Wang C., Li C., Qin Y., Wang Z., Yang F., Li Z., Wang J. (2018). A functional chitosan-based hydrogel as a wound dressing and drug delivery system in the treatment of wound healing. RSC Adv..

[127] Mengistu Lemma S., Bossard F., Rinaudo M. (2016). Preparation of pure and stable chitosan nanofibers by electrospinning in the presence of poly (ethylene oxide). Int. J. Mol. Sci..

[128] Abraham A., Soloman P.A., Rejini V.O. (2016). Preparation of chitosan-polyvinyl alcohol blends and studies on thermal and mechanical properties. Procedia Technol..

[129] Annur D., Wang Z.K., Liao J.D., Kuo C. (2015). Plasma-synthesized silver nanoparticles on electrospun chitosan nanofiber surfaces for antibacterial applications. Biomacromolecules.

[130] Li M., Guo Y., Wei Y., MacDiarmid A.G., Lelkes P.I. (2006). Electrospinning polyaniline-contained gelatin nanofibers for tissue engineering applications. Biomaterials.

[131] Razak A., Izwan S., Wahab I.F., Fadil F., Dahli F.N., Khudzari M., Zahran A., Adeli H. (2015). A review of electrospun conductive polyaniline based nanofiber composites and blends: Processing features, applications, and future directions. Adv. Mater. Sci. Eng..

[132] Ghasemi-Mobarakeh L., Prabhakaran M.P., Morshed M., Nasr-Esfahani M.H., Ramakrishna S. (2009). Electrical stimulation of nerve cells using conductive nanofibrous scaffolds for nerve tissue engineering. Tissue Eng. Part A.

[133] Al-Musawi S., Albukhaty S., Al-Karagoly H., Sulaiman G.M., Alwahibi M.S., Dewir Y.H., Soliman D.A., Rizwana H. (2020). Antibacterial activity of honey/chitosan nanofibers loaded with capsaicin and gold nanoparticles for wound dressing. Molecules.

[134] Liu X.-F., Zhang J., Liu J.-J., Zhou Q.-H., Liu Z., Hu P.-Y., Yuan Z., Ramakrishna S., Yang D.-P., Long Y.-Z. (2020). Bifunctional CuS composite nanofibers via in situ electrospinning for outdoor rapid hemostasis and simultaneous ablating superbug. Chem. Eng. J..

[135] Mouthuy P.A., Groszkowski L., Ye H. (2015). Performances of a portable electrospinningapparatus. Biotechnol. Lett..

[136] Xu S.C., Qin C.C., Yu M., Dong R.H., Yan X., Zhao H., Han W.P., Zhang H.D., Long Y.Z. (2015). A battery-operated portable handheld electrospinning apparatus. Nanoscale.

[137] Haik J., Kornhaber R., Blal B., Harats M. (2017). The feasibility of a handheld electro-spinning device for the application of nanofibrous wound dressings. Adv. Wound Care.

[138] Sadri M., Maleki A., Agend F., Hosseini H. (2017). Retracted: Fast and efficient electro-spinning of chitosan-Poly (ethylene oxide) nanofibers as potential wound dressingagents for tissue engineering. J. Appl. Polym. Sci..

[139] The Stellenbosch Nanofiber Company (SNC). https://www.innovus.co.za/spin-out-companies/the-stellenbosch-nanofiber-company-snc.html.

[140] Bhaskar P., Bosworth L.A., Wong R., O’brien M.A., Kriel H., Smit E., McGrouther D.A., Wong J.K., Cartmell S.H. (2017). Cell response to sterilized electrospun poly (ɛ-caprolactone) scaffolds to aid tendon regeneration in vivo. J. Biomed. Mater. Res. Part A.

[141] Akduman C., Kumbasar E.P.A., Ylmaz F. (2017). Electrospun Polyurethane Nanofibers. Aspects of Polyurethanes.

[142] Knoff A. (2008). Nanofibers Allergen Barrier Fabric. U.S. Patent.

[143] Phonix Nanofibre Acoustic Technology. https://www.revolutionfibres.com/products/phonix/.

[144] Han D., Steckl A. (2019). “Mix-and-Match”: A review of coaxial electrospinning formation of complex polymer fibers and their applications. ChemPlusChem..

[145] Moreira B., de Morais M.G., de Morais E.G., da Silva Vaz B., Costa J.A.V., Grumezescu A.H., Holban A.M. (2018). Electrospun Polymeric Nanofibers in Food Packaging. Impact of Nanoscience in the Food Industry.

[146] Amna T., Yang J., Ryu K.S., Hwang I.H. (2014). Electrospun antimicrobial hybrid mats: Innovative packaging material for meat and meat-products. J. Food Sci. Technol..

[147] Zhu Z., Zhang Y., Shang Y., Wen Y. (2019). Electrospun nanofibers containing TiO_2_ for the photocatalytic degradation of ethylene and delaying postharvest ripening of bananas. Food Bioprocess. Technol..

[148] Yamazaki S., Tanaka S., Tsukamoto H. (1999). Kinetic studies of oxidation of ethylene over a TiO2 photocatalyst. J. Photochem. Photobiol. A Chem..

[149] Maneerat C., Hayata Y. (2006). Efficiency of TiO2 photocatalytic reaction on delay of fruit ripening and removal of off-flavors from the fruit storage atmosphere. Trans. ASAE.

[150] Maneerat C., Hayata Y., Egashira N., Sakamoto K., Hamai Z., Kuroyanagi M. (2003). Photocatalytic reaction of TiO_2_ to decompose ethylene in fruit and vegetables storage. Trans. ASAE.

[151] Wen P., Zhu D.-H., Wu H., Zong M.-H., Jing Y.-R., Han S.-Y. (2016). Encapsulation of cinnamon essential oil in electrospun nanofibrous film for active food packaging. Food Control..

[152] Paniagua A.C., East A.R., Hindmarsh J.P., Heyes J.A. (2013). Moisture loss is the major cause of firmness change during postharvest storage of blueberry. Postharvest Biol. Technol..

[153] Paull R.E., Gross K., Qiu Y. (1999). Changes in papaya cell walls during fruit ripening. Postharvest Biol. Technol..

[154] Vega-Lugo A.C., Lim L.T. (2009). Controlled release of allyl isothiocyanate using soy protein and poly (lactic acid) electrospun fibers. Food Res. Int..

[155] Fathi-Azarbayjani A., Qun L., Chan Y.W., Chan S.Y. (2010). Novel vitamin and gold-loaded nanofiber facial mask for topical delivery. Aaps Pharmscitech.

[156] Camerlo A., Vebert-Nardin C., Rossi R.M., Popa A.M. (2013). Fragrance encapsulation in polymeric matrices by emulsion electrospinning. Eur. Polym. J..

[157] Balamurugan R., Sundarrajan S., Ramakrishna S. (2011). Recent trends in nanofibrous membranes and their suitability for air and water filtrations. Membranes.

[158] Research and Markets: Nanofibers Market Report 2015–2020: The Key Market Trends, Growth Drivers & Challenges. https://www.businesswire.com/news/home/20150922005617/en/Research-and-Markets-Nanofibers-Market-Report-2015–2020-The-Key-Market-Trends-Growth-Drivers-Challenges.

